# Association of Lymphovascular Invasion with Biochemical Recurrence and Adverse Pathological Characteristics of Prostate Cancer: A Systematic Review and Meta-analysis

**DOI:** 10.1016/j.euros.2024.09.007

**Published:** 2024-10-08

**Authors:** Jakub Karwacki, Marcel Stodolak, Andrzej Dłubak, Łukasz Nowak, Adam Gurwin, Kamil Kowalczyk, Paweł Kiełb, Nazar Holdun, Wojciech Szlasa, Wojciech Krajewski, Agnieszka Hałoń, Anna Karwacka, Tomasz Szydełko, Bartosz Małkiewicz

**Affiliations:** a University Center of Excellence in Urology, Department of Minimally Invasive and Robotic Urology, Wroclaw Medical University, Wroclaw, Poland; b Department of Molecular and Cellular Biology, Faculty of Pharmacy, Wroclaw Medical University, Wroclaw, Poland; c Department of Clinical and Experimental Pathology, Wroclaw Medical University, Wroclaw, Poland; d WSB Merito University Wroclaw, Wroclaw, Poland; e University Center of Excellence in Urology, Wroclaw Medical University, Wroclaw, Poland

**Keywords:** Biochemical recurrence, Histopathological examination, Lymphovascular invasion, Prognostic factors, Prostate cancer, Radical prostatectomy, Risk assessment

## Abstract

**Background and objective:**

Lymphovascular invasion (LVI) is a significant histopathological feature in prostate cancer (PCa) associated with higher risk of biochemical recurrence (BCR) and other adverse outcomes. Our aim was to assess the association of LVI found in radical prostatectomy (RP) specimens with BCR and adverse clinicopathological findings.

**Methods:**

A systematic literature search was conducted using the PubMed, Embase, and Web of Science databases in July 2023, with an additional search in May 2024. We included 94 prospective and retrospective studies reporting on LVI in RP specimens and its association with the specified outcomes.

**Key findings and limitations:**

Meta-analyses revealed that LVI is significantly associated with higher BCR risk (hazard ratio 1.96, 95% confidence interval [CI] 1.73–2.21), higher pathological tumour stage (odds ratio [OR] 5.77; 95% CI 3.96–8.40), higher Gleason score (OR 5.19, 95% CI 4.12–6.54), lymph node metastasis (OR 11.52, 95% CI 7.65–17.34), distant metastasis (OR 9.10, 95% CI 5.46–15.17), positive surgical margins (OR 2.38, 95% CI 1.83–3.09), extraprostatic extension (OR 5.01, 95% CI 3.11–8.06), seminal vesicle invasion (OR 7.50, 95% CI 3.47–16.23), and perineural invasion (OR 133.71, 95% CI 65.93–271.15). Major limitations of this study include high heterogeneity of the data and the reliance on nonrandomised studies.

**Conclusions and clinical implications:**

Our findings reveal that LVI is associated with nearly twofold higher risk of BCR, highlighting its potential role as a critical prognostic marker.

**Patient summary:**

We analysed data from multiple studies to understand the impact of the spread of prostate cancer into the lymph or blood vessels, called lymphovascular invasion (LVI). We found that LVI is linked to a higher risk of cancer recurrence after surgery and other negative outcomes. Our findings highlight the importance of considering LVI in treatment decisions for better management of prostate cancer.

## Take Home Message

Lymphovascular invasion (LVI) significantly increases the risk of biochemical recurrence, distant metastasis, and other adverse pathological outcomes in prostate cancer. Incorporation of LVI into clinical decision-making might improve patient management and outcomes.

## 1 Introduction

Prostate cancer (PCa) poses a substantial health burden, ranking as the second most prevalent cancer among men aged ≥50 yr [1]. Lymphovascular invasion (LVI), often defined as the unequivocal presence of tumour cells within endothelium-lined spaces [2], [3], [4] or as the presence of tumour emboli in small intraprostatic vessels [5], [6], has long been recognised as a potential prognostic factor, as it appears to be linked to other adverse histopathological findings and unfavourable oncological outcomes, including biochemical recurrence (BCR) [7], [8], [9], [10]. Despite the recognised importance of LVI, its application in clinical practice remains pending [11]. The European Association of Urology (EAU) guidelines [12] emphasise the integral role of LVI in histopathological assessments following both biopsy and radical prostatectomy (RP). In alignment with the consensus of the International Society of Urological Pathology (ISUP) [13], the EAU guidelines underscore the significance of LVI in PCa and highlight the previous consensus [14] advocating exclusion of patients from active surveillance (AS) if LVI is found in their biopsy specimens. However, despite its notable role in postoperative histopathology, LVI currently has no impact on PCa management in existing guidelines.

The aim of our systematic review and meta-analysis was to explore the association between LVI and BCR, as well as other adverse histopathological findings, including pathological tumour stage (pT), Gleason score (GS), lymph node metastasis (LNM), extraprostatic extension (EPE), perineural invasion (PNI), positive surgical margins (PSMs), seminal vesicle involvement (SVI), and distant metastasis. Considering the dynamic nature of scientific research, an updated and comprehensive evaluation of the current literature is a necessity. Despite its recognition in pathology reports, LVI is still not included in PCa staging because of ongoing debate about its prognostic significance.

## 2 Methods

### 2.1 Search strategy

The investigation adhered to the Preferred Reporting Items for Systematic Reviews and Meta-Analyses (PRISMA) guidelines [15]. The study protocol was preregistered on PROSPERO (International Prospective Register of Systematic Reviews) with the registration number CRD42023395671. In July 2023, a comprehensive systematic search of the PubMed, Embase, and Web of Science databases was independently conducted by three review authors (J.K., M.S., and A.D.). To ensure that the most recent articles were included, an additional brief search was conducted in May 2024. The search criteria included articles in English for which the full text was available, with no time restrictions. The following terms and keywords were used:

• PubMed: “(prostate cancer) AND (microvascular invasion OR lymphovascular invasion)” using Medical Subject Headings (MeSH) terms.
• Embase: “‘prostate cancer’/exp AND (‘lymphovascular invasion’/exp OR ‘microvascular invasion’/exp)“ using Emtree exploded terms.
• Web of Science: “ALL=(prostate cancer AND (lymphovascular invasion OR microvascular invasion))”.

**Fig. 1:**
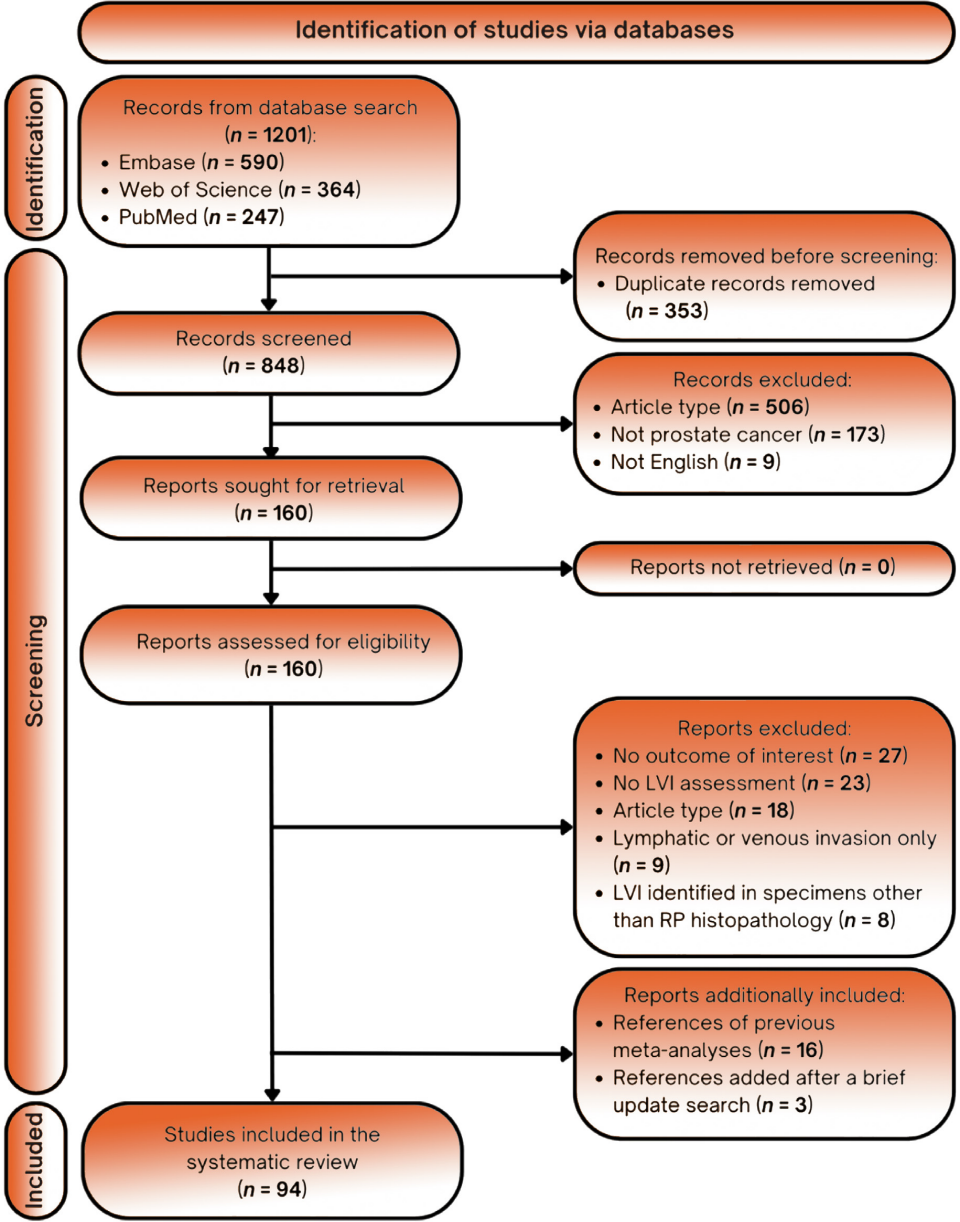
Preferred Reporting Items for Systematic Reviews and Meta-Analyses (PRISMA) flow chart. LVI = lymphovascular invasion; RP = radical prostatectomy.

Reference lists in relevant systematic review articles were also meticulously examined to confirm that no potentially eligible papers were omitted. The study selection process is depicted in Figure 1.

### 2.2 Inclusion and exclusion criteria

Three authors (J.K., B.M., and T.S.) formulated the search strategy and defined the inclusion criteria. The ultimate list of articles to be included was determined via consensus among all the collaborating authors after checking alignment with the inclusion criteria. Studies eligible for included in the systematic review had to meet the following inclusion criteria:

(1) Original investigation;
(2) English language;
(3) Accessibility to the full manuscript;
(4) Patients diagnosed with PCa;
(5) LVI evaluated in RP specimens; and
(6) Associations between LVI and BCR, pathological GS or Gleason grade group (GGG), pT, LNM, EPE, PNI, PSM, SVI, and distant metastasis were evaluated (*p* values, odds ratio [OR], risk ratio [RR], or hazard ratio [HR] extractable).

The exclusion criteria were as follows:

(1) Noncomparative studies, including reviews, letters, conference papers, editorial comments, replies from authors, and case reports;
(2) Studies evaluating LVI in RP specimens with addition of other histopathological samples (eg, LVI assessed in RP and transurethral resection of the prostate specimens); and
(3) Studies not reporting the outcomes of interest.

### 2.3 Study eligibility and quality assessment

Studies were assessed for eligibility using the PICO (Population, Intervention, Comparison, Outcome) approach:

• Population: PCa patients with LVI in final histopathology specimens.
• Intervention: RP and histopathological examination.
• Comparison: PCa patients without LVI in final histopathology specimens.
• Outcome: association of LVI with BCR, pathological GS or GGG, pT, LNM, EPE, PNI, PSM, SVI, or distant metastasis.

The risk of bias (RoB) for each manuscript was evaluated according to the principles outlined in the *Cochrane Handbook for Systematic Reviews of Interventions* [16]. Three authors (J.K., M.S., and W.S.) independently conducted the assessments. The articles were reviewed for their adjustment for major confounders associated with BCR, including age, pT stage, pN stage, pathological GS, preoperative prostate-specific antigen (PSA) levels, and surgical margin status. The risk of confounding bias was deemed high if the confounder was not controlled for in multivariate analysis. Any disagreements or discrepancies were resolved via consensus or consultation with a fourth author (B.M.). RoB assessment was generated using the *robvis* tool [17].

### 2.4 Statistical analysis

All analyses were performed using RevMan v7.9.2 (Cochrane Collaboration, London, UK; https://revman.cochrane.org).

The estimated effects of LVI on BCR risk were calculated using HRs and 95% CIs. The overall pooled HR was estimated by calculating the weighted average of the log[HR] and corresponding 95% CI from each study. An observed HR >1 implies a poor survival outcome for patients with LVI. The statistical significance of the pooled HRs was evaluated using the Z test. Significant heterogeneity was indicated by either a ratio of >50% for the I^2^ statistic or a *p* value of ≤0.05 for Cochran’s Q test. A fixed-effect (FE) model or a random-effect (RE) model was used, depending on the I^2^ value for heterogeneity.

Subgroup analyses were also performed to check whether the pooled HR was influenced by the statistical analysis approach and study setting, number of centres involved, publication date, sample size, mean/median follow-up, mean/median age, mean/median preoperative PSA, percentage of LVI^+^ patients, and the LVI and BCR definitions provided. Cutoff values for different subgroups (eg, median follow-up of 30 mo) were established on the basis of assessment of previous meta-analyses. To assess the stability of the combined HR, sensitivity analysis was performed by removing one study. For each comparison, we conducted sensitivity analysis and assessed for publication bias (visual interpretation of funnel plots).

To determine the significance of LVI for pathological diagnosis, we also investigated associations between LVI and clinicopathological features of PCa. ORs for dichotomous variables were used to calculate a pooled OR with 95% CI. Data for EPE (yes vs no), pathological GS (>7 vs ≤7), LNM (yes vs no), pathological stage (T1–2 vs T3–4), surgical margin status (positive vs negative), distant metastasis (yes vs no), PNI (yes vs no), and SVI (yes vs no) were dichotomised. Event numbers were obtained from the original studies, and ORs and 95% CIs were calculated.

## 3 Results

### 3.1 Study selection and characteristics

The initial search identified 1201 publications. After removal of duplicates, 848 articles were screened by title and abstract, and 688 were excluded. According to the inclusion criteria, we identified 94 studies [2], [3], [5], [6], [18], [19], [20], [21], [22], [23], [24], [25], [26], [27], [28], [29], [30], [31], [32], [33], [34], [35], [36], [37], [38], [39], [40], [41], [42], [43], [44], [45], [46], [47], [48], [49], [50], [51], [52], [53], [54], [55], [56], [57], [58], [59], [60], [61], [62], [63], [64], [65], [66], [67], [68], [69], [70], [71], [72], [73], [74], [75], [76], [77], [78], [79], [80], [81], [82], [83], [84], [85], [86], [87], [88], [89], [90], [91], [92], [93], [94], [95], [96], [97], [98], [99], [100], [101], [102], [103], [104], [105], [106], [107] involving 417 660 patients, of whom 44 453 were LVI-positive (10.6%). Among the studies included, 82 were retrospective [2], [3], [5], [18], [19], [20], [21], [22], [23], [24], [25], [26], [27], [28], [29], [30], [32], [33], [34], [35], [36], [37], [39], [40], [43], [44], [45], [46], [47], [48], [49], [50], [52], [53], [54], [55], [56], [58], [59], [60], [61], [62], [63], [66], [67], [68], [69], [70], [71], [72], [73], [74], [75], [76], [77], [78], [79], [80], [81], [82], [83], [84], [85], [86], [87], [88], [89], [90], [91], [92], [93], [94], [95], [96], [97], [100], [101], [102], [104], [105], [106], [107] and 12 were prospective [6], [31], [38], [41], [42], [51], [57], [64], [65], [98], [99], [103]. In terms of the setting, 67 were single-centre studies [2], [6], [18], [20], [21], [22], [23], [25], [28], [29], [30], [31], [32], [33], [34], [35], [36], [38], [39], [41], [42], [43], [44], [45], [46], [47], [48], [52], [54], [55], [56], [57], [58], [59], [60], [61], [63], [64], [65], [68], [69], [70], [72], [73], [75], [76], [77], [78], [79], [80], [81], [85], [86], [87], [89], [91], [93], [94], [95], [96], [97], [98], [102], [103], [105], [106], [107], 24 were multicentre studies [5], [19], [24], [26], [27], [37], [40], [49], [50], [51], [62], [67], [71], [74], [82], [83], [84], [88], [90], [92], [99], [100], [101], [104], and three were registry-based [3], [53], [66].

The studies included were published between 1998 and 2024. LVI was most commonly defined as the unequivocal presence of tumour cells within endothelium-lined spaces or as the presence of tumour emboli in intraprostatic vessels. However, in most of the studies no definition was provided. The incidence of LVI ranged from 1.4% [33] to 92.6% [18]. The characteristics of the studies are shown in Table 1.

**Table 1:** Study characteristics

Study and country	Design	Recruitment period	Pts	LVI^+^, *n*	Age (yr)	p-PSA	pGS <7/≥7	pT1–2/pT3-4	pN+	NAT	Parameters analysed for LVI association
				(%)		(ng/ml)	(*n*/*n*)	(*n*/*n*)	(*n*)	(*n*)	
Al Qa’qa’ 2022 [18], Canada	RSC	2004–2020	54	50 (92.6)	NA	NA	0/47	5/45	14	NA	pT, LNM, DM
Andersen 2014 [19], Norway	RMC	1995–2005	535	43 (8.0)	62 (45–75)^a^	8.8 (0.7–104.3)^b^	183/352	374/161	3	NA	pT, PNI
Andras 2016 [20], Romania	RSC	2009–2014	105	4 (3.8)	62 (46–74)^b^	Range: 6.9–13.6	33/72	58/47	5	NA	BCR, LNM
Antunes 2006 [21], Brazil	RSC	1993–2000	428	47 (11.0)	62.8 (40–83)^b^	Mean: 10	257/171	309/119	0	0	BCR, pT, GS, PSM, SVI
Ariafar 2021 [22], Iran	RSC	2013–2018	578	70 (12.1)	63.9 ± 6.95^c^	NA	274/304	495/83	31	0	pT, GS
Babaian 2001 [23], USA	RSC	1987–1993	265	NA	64.2 (41–74)^a^	NA	36/229	183/82	0	0	BCR
Bargão Santos 2020 [24], Portugal	RMC	2000–2005	234	NA	64 (46–76)^d^	10.2 (2.2–42.3)^d^	56/175	74/160	NA	NA	BCR
Baydar 2008 [25], Turkey	RSC	1992–2001	71	11 (15.5)	63 (48–75)^a^ Mean: 62	11.5 (1.3–41.5)^b^	18/53	23/48	5	0	pT, GS, EPE, PSM, SVI, LNM
Brooks 2005 [26], USA	RMC	1991–2001	104	11 (10.6)	63 (48–75)^a^	9.9 (1.5–112)^a^	32/65	NA	8	NA	BCR, DM
Brooks 2006 [27], USA	RMC	1991–2001	160	18 (11.3)	NA	Range: 1.3–217	46/101	NA	11	NA	BCR, GS, EPE, PSM, SVI, PNI, LNM, DM
Celik 2020 [28], Turkey	RSC	NA	254	5 (2.0)	NA	Range: 1.4–100	94/155	NA	9	NA	BCR
Chen 2021 [29], USA	RSC	2017–2019	156	34 (21.8)	66 (61–70)^d^	8.0 (5.7–11.9)^d^	0/156	61/95	17	NA	BCR
Cheng 2005 [30], USA	RSC	1990–1998	504	106 (21.0)	63 (34–80)^a^ Mean: 62	NA	182/322	348/156	18	0	BCR, pT, GS, EPE, PSM, SVI, PNI, LNM
Cho 2010 [31], South Korea	PSC	2005–2009	167	16 (9.6)	64.4 (49–80)^b^	9.8 (0.8–79)^b^	NA	126/45	NA	0	BCR
Chromecki 2011 [32], USA	RSC	NA	110	8 (7.3)	62.6 (9.2)^d^	7.7 (5.5)^d^	102/128	NA	6	0	BCR, DM
Chung 2018 [33], South Korea	RSC	2010–2015	213	3 (1.4)	64 (59–69)^d^	5.4 (4.0–7.5)^d^	115/98	185/28	NA	0	BCR
de la Taille 2000 [34], USA	RSC	1993–1998	241	30 (12.4)	62 (42–77)^b^	7.4 (1.2–35)^b^	121/120	165/76	NA	NA	BCR, pT, GS, EPE, PSM, SVI
Dere 2017 [35], Turkey	RSC	2001–2013	117	10 (8.5)	67 (46–81)^a^	8.2 (1.7–72)^a^	56/61	78/39	NA	NA	BCR, pT
Epstein 2000 [36], USA	RSC	1984–1994	60	13 (21.7)	NA	NA	4/56	NA	0	0	BCR
Fajkovic 2016 [37], multicentre	RMC	2000–2011	6678	767 (11.5)	61 (57–66)^d^	6 (4–9)^d^	2197/4489	NA	0	0	BCR, GS, EPE, PSM, SVI
Ferrari 2004 [38], USA	PSC	1984–1999	620	110 (17.7)	NA	NA	113/501	391/229	39	0	BCR, GS, EPE, PSM, SVI, LNM
Fujimura 2017 [39], Japan	RSC	2005–2016	908	282 (31.1)	Median: 67	Range: 1.3–77	345/562	650/258	10	0	BCR
Furukawa 2016 [40], Japan	RMC	2004–2013	382	149 (39.0)	67.8 (50–79)^a^	15.9 (2.9–65.4)^a^	75/307	227/155	21	0	BCR
Galiabovitch 2016 [41], Australia	PSC	2004–2012	1267	82 (6.5)	NA	NA	145/1165	908/402	NA	NA	BCR, pT, GS, PSM
Gesztes 2022 [42], USA	PSC	1993–2013	188	50 (26.6)	60.4 ± 7.3^c^	5.8 (0.4–94.2)^a^	NA	116/73	NA	0	pT, GS, PSM, DM
Goenka 2012 [43], USA	RSC	1988–2007	285	57 (20.0)	61 (40–74)^a^	8.2 (0.9–252)^a^	38/247	NA	17	84	BCR
Gottlieb 2023 [44], USA	RSC	2015–2021	66	39 (59.1)	65.6 ± 7.4^c^	13.2 ± 12.3^c^	NA	14/52	66	0	LNM
Gun 2021 [45], Turkey	RSC	2009–2017	285	23 (8.1)	63.48 ± 6.72^c^ Range: 45–84	7.8 (5.5–12.3)^a^ Range: 2.2–61	144/141	198/87	NA	NA	BCR
Hashimoto 2020 [46], Japan	RSC	2000–2018	550	298 (54.2)	66.0 ± 6.3^c^	14.8 ± 13.3^c^	32/518	256/294	32	0	BCR
Hashimoto 2014 [47], Japan	RSC	2006–2013	784	176 (22.4)	64.3 (60–69)^e^	8.9 (5.1–9.7)^e^	63/721	625/157	6	0	BCR
Herman 2000 [48], USA	RSC	1983–1997	263	91 (34.6)	mean: 64	NA	73/192	0/263	0	8	GS, EPE, SVI
Hong 2017 [49], USA	RMC	2006–2014	205	33 (16.1)	61.6 ± 6.9^c^ 62 (57.0–67.0)^d^	6.3 (4.5–8.9)^d^	57/148	0/205	0	0	BCR
Hsieh 2022 [50], Taiwan	RMC	2012–2017	579	97 (16.8)	NA	NA	NA	306/273	49	NA	BCR
Huang 2007 [51], Taiwan	PMC	2000–2005	126	NA	NA	NA	NA	68/51	7	NA	BCR
Ito 2003 [52], Japan	RSC	1989–1998	82	38 (46.3)	66.5 ± 0.5^c^ Range: 56–74	17.2 ± 1.8^c^ Range: 0.6–110	35/47	50/32	0	0	BCR, pT, GS, EPE, PSM, SVI, PNI
Jamil 2021 [53], USA	RRB	2010–2015	232 704	17,758 (7.6)	62 (56–67)^e^	5.6 (4.3–8.2)^e^	63 631/164 941	174,838/57,866	6129	NA	pT, GS, LNM
Jeon 2009 [54], South Korea	RSC	1995–2004	237	41 (17.3)	64.5 (44–86)^a^	11.5 (0.2–98)^a^	52/183	145/92	5	0	BCR, pT, GS, EPE, PSM, SVI
Jeong 2017 [55] South Korea	RSC	1995–2015	61	26 (42.6)	68 ± 5.6^c^ 68 (51–77)^a^	11.7 ± 10.3^c^ 11.7 (0.6–66.4)^a^	0/61	17/44	3	0	BCR
Jeong 2019 [56], South Korea	RSC	2006–2015	168	25 (14.9)	68 (50–78)^a^	14.9 (2.1–177.0)^a^	10/154	54/111	9	NA	DM
Joung 2007 [57], South Korea	PSC	2005–2006	66	9 (13.6)	65.2 (49–80)^b^	23.3 (3.7–98.3)^b^	31/35	39/25	NA	22	BCR
Jung 2011 [58], South Korea	RSC	2005–2009	407	27 (6.6)	63.2 (38–82)^b^	10.0 (2.8–83.2)^b^	160/247	282/125	12	0	BCR, pT GS, EPE, PSM, SVI, LNM
Kamitani 2020 [59], Japan	RSC	1997–2018	176	37 (21.0)	66 ± 6^c^	Median: 9.0	0/176	77/99	7	0	BCR, GS
Kang 2017 [60], South Korea	RSC	2005–2014	1600	118 (7.4)	66 (61–71)^d^	8.2 (5.3–15.5)^d^	434/1166	741/858	45	NA	BCR, LNM, DM
Kang 2016 [61], South Korea	RSC	2003–2014	2034	252 (12.4)	NA	NA	308/1726	1481/ 553	20	0	BCR, pT GS, EPE, PSM, SVI, LNM
Karwacki 2024 [4], Poland	RSC	2012–2021	861	152 (17.7)	64.1 (31–80)^b^	14.0 (0–174)^b^	122/739	493/368	143	NA	pT, GS, EPE, PSM, PNI, LNM
Kawase 2024 [62], Japan	RMC	2012–2021	2608	770 (29.5)	NA	NA	204/2398	1895/713	0	0	BCR, pT, GS, PSM, DM
Kim 2021 [63], South Korea	RSC	1997–2017	389	59 (15.2)	NA	NA	0/389	223/166	33	0	BCR
Kim 2015 [64], South Korea	PSC	2005–2012	110	NA	NA	mean: 11.7 ± 10.3	NA	NA	1	NA	BCR
Kneebone 2017 [65], Australia	PSC	2008–2013	156	36 (23.1)	NA	NA	NA	60/129	13	NA	BCR
Koparal 2021 [66], Turkey	RRB	NA	984	48 (4.9)	Range: 30–83	Range: 0.7–87.0	NA	NA	31	NA	BCR
Kozal 2015 [6], France	PSC	2005–2013	742	21 (2.8)	62.3 ± 6.9^c^	8.4 ± 6.1^c^	271/471	538/204	19	0	BCR
Lee 2010 [67], South Korea	RMC	1999–2010	361	40 (11.1)	69 ± 6.8^c^ Range: 49–94	15.6 ± 18.6^c^	144/217	253/108	13	0	BCR
Leng 2013 [68], South Korea	RSC	2005–2010	166	40 (24.1)	NA	NA	0/166	109/57	NA	0	BCR
Liauw 2003 [69], USA	RSC	1988–2000	15	5 (33.3)	59 (46–79)^a^	NA	NA	23/28	4	NA	BCR
Loeb 2006 [5], USA	RMC	1989–2004	1709	118 (6.9)	NA	NA	1166/543	NA	11	NA	BCR, pT, GS, PSM, SVI, LNM,
Luo 2012 [70], Taiwan	RSC	1998–2010	87	18 (20.7)	63 (49–83)^b^	NA	70/17	NA	5	NA	BCR, GS, EPE, PSM, SVI, LNM
May 2007 [71], Germany	RMC	1996–2003	412	42 (10.2)	63.7 (44–79)^b^	12.1 (0.1–151)^b^	243/169	299/113	0	0	BCR, pT GS, PSM
Mian 2002 [72], USA	RSC	1987–1998	188	NA	63 (48–73)^a^	8.6 (1.6–42)^a^	0/188	71/117	11	0	BCR
Micoogullari 2021 [73], Turkey	RSC	2009–2017	857	86 (10.0)	NA	NA	393/482	562/313	52	0	BCR
Mitsuzuka 2015 [74], Japan	RMC	2000–2009	1144	120 (10.5)	NA	NA	157/1003	796/364	28	0	BCR, pT, GS, PSM, LNM
Miyai 2014 [75], USA	RSC	2006–2012	901	23 (2.6)	NA	NA	NA	NA	22	0	BCR
Mizuno 2006 [76], Japan	RSC	1997–2001	164	45 (27.4)	65.9 (52–74)^b^	12.5 (1.6–53.6)^b^	NA	NA	NA	0	EPE, SVI
Mizuno 2009 [77], Japan	RSC	NA	164	44 (26.8)	65.6 (52–74)^b^	Mean: 11.5 Median: 11.5	NA	102/62	NA	0	BCR
Numbere 2022 [78], USA	RSC	2009–2018	248	70 (28.2)	NA	NA	NA	0/248	93	0	EPE
Ohno 2016 [79], Japan	RSC	2002–2010	562	148 (26.3)	65.9 ± 6.4^c^	10.6 ± 10.1^c^	100/462	NA	7	0	BCR
Oufattole 2023 [80], USA	RSC	2005–2020	130	45 (34.6)	65 (43–79)^a^	7.9 (1.1–43.1)^a^	0/130	27/103	5	0	BCR
Özkanli 2014 [81], Turkey	RSC	2001–2010	94	30 (31.9)	62.81 ± 6.87^c^ Range: 42–73	NA	NA	NA	0	0	BCR, PSM
Özsoy 2018 [82], multicentre	RMC	2000–2011	6041	693 (11.5)	61 (57–66)^d^	6 (4–9)^d^	1932/4109	NA	116	0	BCR, GS
Pagano 2015 [83], USA	RMC	1990–2011	180	75 (41.7)	63.7 (58.8–67.6)^d^	9.1 (6.3–17.1)^d^	90/90	67/113	22	NA	BCR
Park 2016 [84], South Korea	RMC	2001–2012	1209	260 (21.5)	66.2 ± 6.5^c^ Median: 67	15.8 ± 17.8^c^ Median: 10.9	85/1122	31/1179	0	0	BCR
Psutka 2011 [85], USA	RSC	1993–1995	300	NA	NA	NA	NA	249/51	0	0	PSM
Quinn 2001 [86], Australia	RSC	1986–1999	731	38 (5.2)	62.7 (40.7–76.7)^a^ Mean: 62.1	9 (0.7–194)^a^ Mean: 13	389/343	407/308	17	0	BCR
Rakic 2021 [3], USA	RRB	2010–2015	126,882	12,632 (10.0)	62 (57–66)^d^	5.9 (4.5–8.9)^d^	23 863/102 819	87 021/39 661	5010	NA	pT, GS, PSM
Rodrigues 2021 [87], Portugal	RSC	2012–2017	199	42 (21.1)	68 (48–81)^a^	8.2 (2.1–80.2)^a^	NA	156/117	4	NA	BCR
Safdieh 2014 [88], USA	RMC	2003–2010	52	6 (11.5)	NA	NA	10/40	31/21	NA	0	BCR
Salomao 1995 [89], USA	RSC	1991–1992	210	111 (52.9)	NA	NA	66/144	114/77	19	0	GS, EPE, PSM, SVI, LNM
Sathianathen 2023 [90], Australia	RMC	1994–2021	3495	653 (18.7)	63 (58–68)^d^	7 (5–11)^d^	608/2887	3055/440	292	0	BCR, pT, GS, PSM, LNM, DM
Sertkaya 2014 [91], Turkey	RSC	2004–2011	167	15 (9.0)	66.4 ± 12.3^c^	6.7 ± 3.1^c^ Range: 0.2–10.0	NA	122/45	NA	NA	EPE
Sevcenco 2016 [92], multicentre	RMC	2000–2011	7205	6299 (87.4)	61 (57–66)^d^	6 (4–9)^d^	2165/5040	1944/5261	668	0	BCR
Shariat 2004 [2], USA	RSC	1994–2002	630	32 (5.1)	60.4 ± 6.7^c^ 60.9 (40–75)^a^	8.1 ± 8^c^ 6.1 (4.5–8.7)^d^ Range: 0.1–99	256/374	NA	10	NA	BCR, GS, EPE, PSM, SVI, LNM, DM
Shin 2021 [93], South Korea	RSC	2009–2016	214	14 (6.5)	NA	NA	36/178	214/0	0	NA	BCR
Stamey 2000 [94], USA	RSC	1983–1992	326	NA	65 (60–69)^d^ 64 (35–79)^b^	7.3 (4.2–12.5)^d^ 11.1 (0.3–146.3)^b^	NA	NA	31	0	BCR
Taguchi 2016 [95], Japan	RSC	2003–2014	116	35 (30.2)	66 (61–71)^d^	9 (6.3–12.3)^d^	27/89	63/53	0	0	BCR
Taverna 2015 [96], Italy	RSC	1999–2004	70	9 (12.9)	62 ± 6^c^	6.5 ± 0.2^c^	44/26	62/8	1	NA	GS
Tokuda 2010 [97], USA	RSC	NA	115	74 (64.3)	NA	NA	NA	16/109	125	13	LNM
van den Ouden 1998 [98], Netherlands	PSC	1977–1994	273	33 (12.1)	63.8 (45–75)^a^	Range: 0–181.4	NA	86/187	27	0	pT, EPE, PSM, SVI, PNI, LNM, DM
Vau 2019 [99], Portugal	PMC	2012–2016	144	7 (4.9)	61.4 ± 5.6^c^ Range: 47–75	8.1 ± 4.9^c^ Range: 1–23	19/125	82/62	8	0	BCR, GS
Wessels 2021 [100], Germany	RMC	NA	218	109 (50.0)	68 (64–73)^d^	12.0 (7.3–21)^d^	0/218	20/198	102	NA	LNM
Whittemore 2008 [101], USA	RMC	1988–2003	214	12 (5.6)	NA	NA	0/214	163/51	5	0	BCR, GS
Yamamoto 2008 [102], Japan	RSC	1994–2005	94	26 (27.7)	68 (52–76)^d^	9.7 1.7–75.0)^d^	29/65	0/94	0	0	BCR, GS, PSM, DM
Yee 2010 [103], USA	PSC	2004–2007	1298	129 (9.9)	Median: 59	Median: 5.3	320/978	820/375	94	0	BCR, pT, GS, EPE, PSM, SVI, LNM
Yoneda 2018 [104], multicentre	RMC	2010–2014	238	32 (13.4)	67.8 (50–76)^a^	9.0 (2.3–34.9)^a^	NA	183/50	4	0	BCR
You 2014 [105], South Korea	RSC	2000–2009	397	74 (18.6)	64.7 ± 6.3^c^	14.2 ± 13.2^c^	32/365	0/397	0	0	BCR, PSM
Yuksel 2017 [106], Turkey	RSC	2011–2016	62	19 (30.6)	NA	NA	23/39	33/29	4	0	BCR, PSM

Pts = patients; PMC = prospective multicentre study; PSC = prospective single-centre study; RMC = retrospective multicentre study; RRB = retrospective registry-based study; RSC = retrospective single-centre study; LVI = lymphovascular invasion; p-PSA = preoperative prostate-specific antigen; pGS = pathological Gleason score; pT = pathological tumour stage; pN = pathological nodal stage; NA = not available; LNM = lymph node metastasis; DM = distant metastasis; PNI = perineural invasion; BCR = biochemical recurrence; PSM = positive surgical margin; SVI = seminal vesicle invasion; EPE = extraprostatic extension; NAT = neoadjuvant therapy.

a Median (range).

b Mean (range).

c Mean ± standard deviation.

d Median (interquartile range),

e Mean (interquartile range.

### 3.2 RoB and quality assessment

The RoB assessment for the studies included is outlined in Supplementary Figures 1 and 2. Following the principles of the Cochrane Handbook for Systematic Reviews of Interventions, the evaluation for each manuscript considered allocation; sequence generation and concealment; blinding of participants, personnel, and outcome assessors; completeness of the outcome data; selective outcome reporting; other potential sources of bias; and major confounders affecting BCR (age, pT stage, pN stage, pathological GS, preoperative PSA, and surgical margin status). All the studies had a nonrandomised design.

### 3.3 Meta-analyses

We performed meta-analyses for the association of LVI with BCR, pathological GS (or GGG), pT stage, LNM, EPE, PNI, PSM, SVI, and distant metastasis. The meta-analysis results for the association of LVI with adverse histopathological outcomes are summarised in Table 2.

**Table 2:** Summary of meta-analysis results for the associations of lymphovascular invasion with other adverse clinicopathological findings

Variable	Studies, *n* (participants)	Heterogeneity		Effect model	Pooled OR (95% CI)	*p* value
		I^2^ (%)	*p* value			
pT stage (≥T3 vs <T3)	16 (378 409)	99	<0.00001	RE	5.77 (3.96–8.40)	<0.00001
Pathological GS (>7 vs ≤7)	24 (396 507)	97	<0.00001	RE	5.19 (4.12–6.54)	<0.00001
LNM (yes vs no)	15 (153 919)	85	<0.00001	RE	11.52 (7.65–17.34)	<0.00001
Distant metastasis (yes vs no)	3 (2956)	0	0.99	FE	9.10 (5.46–15.17)	<0.00001
PSM (yes vs no)	25 (148 134)	93	<0.00001	RE	2.38 (1.83–3.09)	<0.00001
EPE (yes vs no)	18 (13 275)	90	<0.00001	RE	5.01 (3.11–8.06)	<0.00001
SVI (yes vs no)	16 (12 323)	95	<0.00001	RE	7.50 (3.47–16.23)	<0.00001
Perineural invasion (yes vs no)	4 (1764)	0	0.6	FE	133.71 (65.93–271.15)	<0.00001

OR = odds ratio; CI = confidence interval; GS = Gleason score; LNM = lymph node metastasis; PSM = positive surgical margin; EPE = extraprostatic extension; SVI = seminal vesicle invasion; RE = random effect; FE = fixed effect.

#### 3.3.1 Biochemical recurrence

**Fig. 2:**
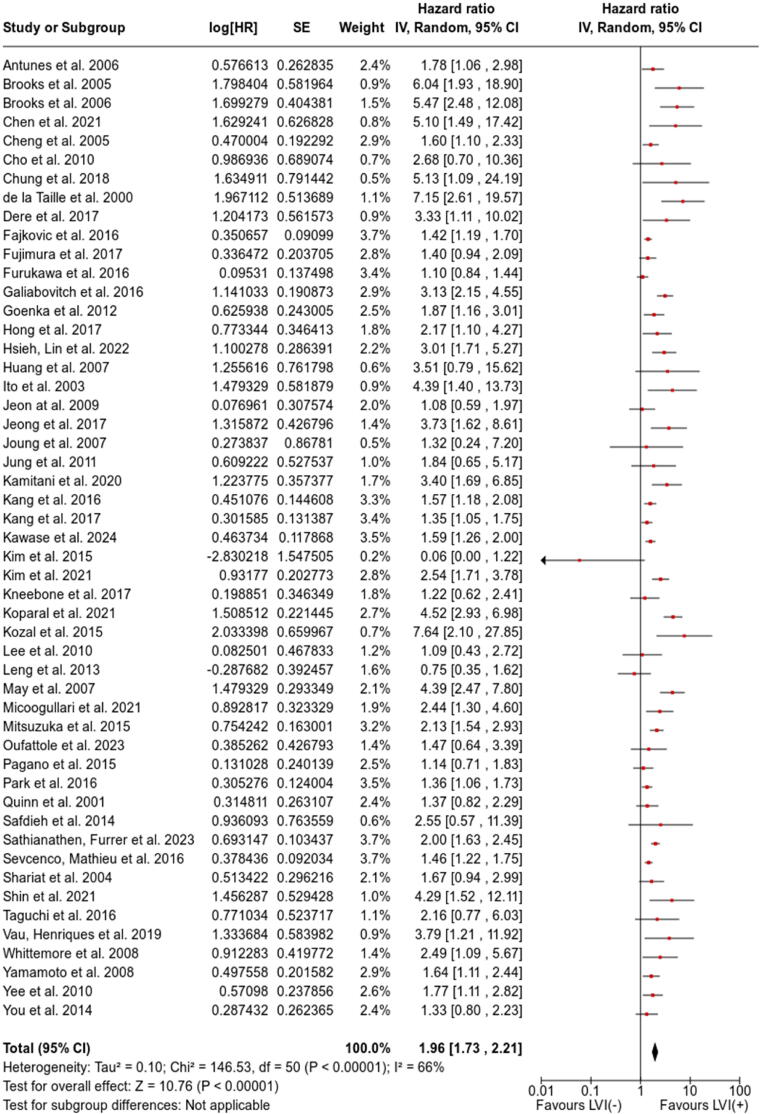
Forest plot and meta-analysis of studies evaluating the association between LVI and the risk of biochemical recurrence for men who underwent radical prostatectomy. HR = hazard ratio; SE = standard error; IV = inverse variance; CI = confidence interval; df = degrees of freedom; LVI = lymphovascular invasion.

**Fig. 3:**
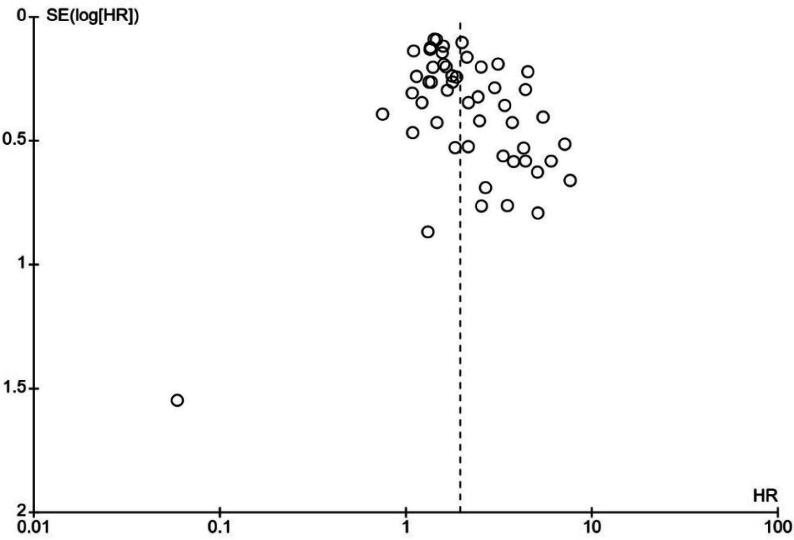
Funnel plot for evaluation of potential publication bias in 51 studies included in the main meta-analysis of biochemical recurrence. SE = standard error; HR = hazard ratio.

There were 51 studies with extractable data on the association between LVI and BCR (HR and CI or HR and *p* value). A forest plot is presented in Figure 2. LVI on final histopathological examination was associated with higher BCR risk (HR 1.96, 95% CI 1.73–2.21; *p* < 0.00001). Owing to high heterogeneity for the data, we performed subgroup analyses, the results of which are presented in Table 3. Forest plots for subgroup analyses are available in the Supplementary Figures 3–13). Examination of the funnel plot revealed significant publication bias (Fig. 3). However, the results were difficult to interpret because of the lack of CI lines in the plot, as CI lines are not available when an RE model is applied. A funnel plot for FE model application is presented in Supplementary Figure 22.

**Table 3:** Summary of subgroup analyses for the association of LVI with biochemical recurrence

Analysis specification	Studies	Heterogeneity		Effect model	Pooled HR	*p* value
	(*n*)	I^2^ (%)	*p* value		(95% CI)	
Overall	51	66	<0.00001	RE	1.96 (1.73–2.21)	<0.00001
Statistical analysis approach						
Multivariate	45	63	<0.00001	RE	1.89 (1.66–2.14)	<0.00001
Univariate	6	71	0.004	RE	2.48 (1.63–3.78)	<0.0001
Study setting						
Prospective	9	54	0.02	RE	2.29 (1.45–3.60)	0.0004
Retrospective	42	66	<0.00001	RE	1.91 (1.69–2.17)	<0.00001
Number of centres						
Multicentre/registry-based	19	77	<0.00001	RE	2.02 (1.67–2.45)	<0.00001
Single centre	32	53	0.0003	RE	1.92 (1.63–2.25)	<0.00001
Publication date						
≥2016	25	72	<0.00001	RE	1.97 (1.68–2.30)	<0.00001
<2016	26	58	0.0001	RE	1.95 (1.59–2.40)	<0.00001
Sample size						
≥500 patients	18	71	<0.00001	RE	1.82 (1.58–2.10)	<0.00001
<500 patients	33	62	<0.00001	RE	2.16 (1.74–2.67)	<0.00001
Mean/median follow-up						
≥30 mo	29	68	<0.00001	RE	1.89 (1.60–2.23)	<0.00001
<30 mo	16	70	<0.00001	RE	2.29 (1.75–3.00)	<0.00001
Mean/median age						
≥65 yr	15	51	0.01	RE	1.67 (1.35–2.06)	<0.00001
<65 yr	23	66	<0.00001	RE	2.03 (1.70–2.42)	<0.00001
Mean/median p-PSA						
≥10 ng/ml	17	71	<0.00001	RE	1.86 (1.45–2.40)	<0.00001
<10 ng/ml	20	57	0.001	RE	1.94 (1.63–2.30)	<0.00001
LVI definition provided						
Yes	25	65	<0.00001	RE	1.91 (1.64–2.21)	<0.00001
No	26	68	<0.00001	RE	2.03 (1.63–2.52)	<0.00001
Percentage of LVI^+^ patients						
≥15%	23	56	0.0006	RE	1.66 (1.44–1.90)	<0.00001
<15%	26	70	<0.00001	RE	2.41 (1.96–2.97)	<0.00001
BCR definition						
PSA >0.1 ng/ml	3	58	0.10	RE	2.02 (1.23–3.33)	0.006
PSA >0.2 ng/ml	41	70	<0.00001	RE	2.05 (1.78–2.36)	<0.00001
PSA nadir + 0.2 ng/ml	2	0	0.78	RE	1.33 (1.05–1.70)	0.02
PSA >0.4 ng/ml	4	0	0.77	RE	1.61 (1.14–2.27)	0.007

HR = hazard ratio; CI = confidence interval; RE = random effect; BCR = biochemical recurrence; p-PSA = preoperative prostate-specific antigen; LVI = lymphovascular invasion.

#### 3.3.2 Pathological tumour stage

The association between LVI and pT stage was analysed in 24 studies [3], [5], [18], [19], [21], [22], [25], [30], [34], [35], [41], [42], [52], [53], [54], [58], [61], [62], [71], [74], [90], [98], [103], [107] of which 16 [3], [21], [25], [30], [34], [35], [41], [53], [54], [58], [62], [71], [74], [98], [103], [107] had extractable data that were subsequently dichotomised (pT1–2 vs pT3–4). Of the 378 409 patients, 32 901 (8.7%) were LVI-positive. Patients with LVI were at higher risk of having higher pT stage (OR 5.77, 95% CI 3.96–8.40; *p* < 0.00001).

#### 3.3.3 Pathological Gleason score

The association between LVI and either GS or the GGG system was analysed in 33 studies [2], [3], [5], [21], [22], [25], [27], [30], [34], [37], [38], [41], [42], [48], [52], [53], [54], [58], [59], [61], [62], [70], [71], [74], [82], [89], [90], [96], [99], [101], [102], [103], [107]. The data were dichotomised (GS >7 vs GS ≤7); 24 studies [2], [3], [21], [25], [27], [30], [37], [38], [41], [42], [48], [53], [54], [58], [61], [62], [71], [74], [82], [89], [90], [99], [103], [107] had extractable data and were included in the meta-analysis. Of the 396 507 patients analysed, 34 230 (8.6%) were LVI-positive. Patients with LVI were at higher risk of having higher pathological GS (OR 5.19, 95% CI 4.12–6.54; *p* < 0.00001).

#### 3.3.4 Lymph node metastasis

The association between LVI and LNM was analysed in 21 studies [2], [5], [18], [20], [25], [27], [30], [38], [44], [53], [58], [60], [61], [70], [74], [89], [90], [98], [100], [103], [107] of which 15 [2], [25], [27], [30], [38], [53], [58], [70], [74], [89], [90], [98], [100], [103], [107] had extractable data (number of LVI-positive and LVI-negative patients with and without nodal involvement) and were included in the meta-analysis. Of the 153 919 patients analysed, 15 942 (10.4%) were LVI-positive. Patients with LVI were at higher risk of nodal involvement (OR 11.52, 95% CI 7.65–17.34; *p* < 0.00001).

#### 3.3.5 Distant metastasis

The association between LVI and distant metastasis was analysed in 12 studies [2], [18], [26], [27], [32], [42], [56], [60], [62], [90], [98], [102], of which one [18] did not differentiate between distant metastasis and nodal involvement. Among the remaining 11 studies, three [27], [42], [62] provided extractable data on the numbers of LVI-positive and LVI-negative patients with and without distant metastasis. Median follow-up was 8.3 yr [27] and 13.0 yr [42] for two overall cohorts. In the study by Kawase et al [62], median follow-up for 23.5 mo for the LVI-negative group and 29.4 mo for the LVI-positive group. Of the 2956 patients analysed in the three eligible studies, 838 (28.3%) were LVI-positive. Patients with LVI were at higher risk of distant metastasis (OR 9.10, 95% CI 5.46–15.17; *p* < 0.00001).

#### 3.3.6 Positive surgical margins

The association of LVI and surgical margin status was analysed in 30 studies [2], [3], [5], [21], [25], [27], [30], [34], [37], [38], [41], [42], [52], [54], [58], [61], [62], [70], [71], [74], [81], [85], [89], [90], [98], [102], [103], [105], [106], [107] of which 25 [2], [3], [21], [25], [27], [30], [34], [37], [38], [41], [42], [54], [58], [62], [70], [71], [74], [81], [89], [90], [98], [102], [103], [106], [107] had extractable data (number of LVI-positive and LVI-negative patients with positive and negative surgical margins) and were included in the meta-analysis. Of the 148 134 patients analysed, 16 942 (11.4%) were LVI-positive. Patients with LVI were at higher risk of PSM status (OR 2.38, 95% CI 1.83–3.09; *p* < 0.00001).

#### 3.3.7 Extraprostatic extension

The association of LVI and EPE was analysed in 21 studies [2], [21], [25], [27], [30], [34], [37], [38], [48], [52], [54], [58], [61], [70], [76], [78], [89], [91], [98], [103], [107], of which 18 [2], [21], [25], [27], [30], [34], [37], [38], [54], [58], [70], [76], [78], [89], [91], [98], [103], [107] had extractable data (number of LVI-positive and LVI-negative patients with and without EPE) and were included in the meta-analysis. Of the 13 275 patients analysed, 1761 (13.3%) were LVI-positive. Patients with LVI were at higher risk of EPE (OR 5.01, 95% CI 3.11–8.06; *p* < 0.00001).

#### 3.3.8 Seminal vesicle invasion

The association between LVI and SVI was analysed in 19 studies [2], [5], [21], [25], [27], [30], [34], [37], [38], [48], [52], [54], [58], [61], [70], [76], [89], [98], [103]. Of these, 16 [2], [21], [25], [27], [30], [34], [37], [38], [48], [54], [58], [70], [76], [89], [98], [103] had extractable data (number of LVI-positive and LVI-negative patients with and without SVI) and were included in the meta-analysis. Of the 12 323 patients analysed, 1677 (13.6%) were LVI-positive. Patients with LVI were at higher risk of SVI (OR 7.50, 95% CI 3.47–16.23; *p* < 0.00001).

#### 3.3.9 Perineural invasion

Six studies [19], [27], [30], [52], [98], [107] analysed the association between LVI and PNI. Of these, four studies [27], [30], [98], [107] had extractable data, making them eligible for inclusion in the meta-analysis. Of the 1764 patients analysed, 301 (17.1%) were LVI-positive. Patients with LVI were at higher risk of PNI (OR 133.71, 95% CI 65.93–271.15; *p* < 0.00001).

## 4 Discussion

Our systematic review and meta-analysis explored the association between LVI and BCR, as well as other adverse pathological characteristics in PCa. This study is distinguished by its comprehensive inclusion of studies, encompassing a total of 94 articles with data for 417 660 patients.

The meta-analyses results indicate a strong association between LVI and adverse oncological outcomes, including BCR. Specifically, LVI was associated with a nearly doubled risk of BCR (pooled HR 1.96, 95% CI 1.73–2.21; *p* < 0.00001), highlighting its role as a significant prognostic factor in clinical settings. Importantly, when only multivariate analyses were included in the subgroup analysis, the meta-analysis revealed a statistically significant association, with a HR of 1.89 (95% CI 1.66–2.14; *p* < 0.00001). Our analysis also revealed a marked association between LVI and distant metastasis (OR 9.10, 95% CI 5.46–15.17; *p* < 0.00001), which emphasises the aggressive nature of PCa with LVI on final histopathology.

Interestingly, our subgroup analysis revealed that when the percentage of LVI-positive patients was <15%, the HR for BCR increased significantly. Specifically, studies in which LVI-positive patients accounted for <15% of the sample population had a pooled HR of 2.41 (95% CI 1.96–2.97; *p* < 0.00001) for BCR, in comparison to a pooled HR of 1.66 (95% CI 1.44–1.90; *p* < 0.00001) for studies with a higher percentage of LVI-positive men. This suggests that in populations or centres with lower LVI prevalence, the presence of LVI may be a more potent predictor of recurrence. This could be because LVI represents a particularly aggressive disease phenotype in such populations, thereby markedly influencing the risk of BCR. These findings underscore the importance of considering the prevalence of LVI in patient cohorts when evaluating its prognostic significance, and further support the integration of LVI status into individualised patient risk assessments and treatment planning. This finding also aligns with the study by Galiabovitch et al [41], which suggested the existence of a “grey area” for LVI detection whereby the presence of LVI (“equivocal” LVI) might be identified by one pathologist but overlooked by another, leading to variability in LVI reporting. Such discrepancies can significantly impact the perceived prevalence of LVI in different studies and subsequently affect the prognostic value attributed to LVI. This highlights the need for standardised criteria and training in the identification of LVI to reduce variability and improve the consistency of histopathological evaluations.

Previous systematic reviews and meta-analyses have similarly reported the prognostic significance of LVI in PCa [7], [8], [9], [10]. However, our study is distinct in its inclusion of a larger number of studies and a more recent data set. This comprehensive approach not only corroborates earlier findings but also strengthens the evidence base, making our meta-analysis a pivotal reference point for future research. Moreover, unlike prior analyses, our study investigated the association of LVI with distant metastases and PNI, providing novel insights into these aspects. Taking all these points into account, this meta-analysis now represents the strongest scientific evidence on the role of LVI in PCa.

Despite the recognised importance of LVI in pathology reports, its integration into clinical practice and PCa staging remains limited. Current guidelines, including those from the EAU, emphasise the need for LVI assessment but stop short of incorporating it in treatment decision algorithms. This omission may be because of variability in LVI detection and reporting, a lack of consensus on its independent prognostic value, a limited impact on existing treatment decisions, and insufficient large-scale studies.

Our findings suggest that LVI should play a more prominent role in prognostic models and treatment planning, particularly for adjuvant therapies. LVI incorporation in risk stratification could identify patients who might benefit from more aggressive treatments, such as radiation or androgen deprivation therapy, and inform decisions on intensified surveillance. In addition, LVI integration in prognostic models could improve the accuracy of outcome predictions and support more personalised patient management.

Several limitations of our analysis must be acknowledged. First, the retrospective nature of the majority of the studies included (82 out of 94) may have introduced biases inherent to such a design, including selection bias and unmeasured confounders. Second, there was significant heterogeneity across the studies regarding the definitions of LVI and BCR, which could affect the generalisability of our results. Third, while we used rigorous methodologies to assess RoB and study quality, inherent limitations of the primary studies could still have an influence on our findings. Finally, despite our extensive search strategy, some relevant studies might have been missed, with potential impact on the comprehensiveness of our analysis.

While our study focused on LVI in RP specimens, it is important to acknowledge the challenges and implications of detecting LVI in biopsy specimens. LVI is more difficult to identify in prostate biopsies because of the limited tissue sampling and the focal nature of the invasion, which can lead to underdetection in comparison to RP specimens. As noted in the literature, LVI in needle biopsies is an unusual finding, even in high-grade or high-volume disease [108]. Future studies should consider evaluating LVI in both RP and biopsy specimens to better understand its role in early prognostic assessments and its potential impact on preoperative decision-making.

Furthermore, future research should aim to standardise the definition and assessment of LVI in PCa to reduce heterogeneity and enhance the comparability of studies. Prospective and randomised studies are particularly needed to validate our findings and further elucidate the role of LVI in PCa prognosis. In addition, integration of LVI into clinical practice guidelines could be explored, particularly for stratifying patients for receipt of adjuvant therapies.

## 5 Conclusions

This meta-analysis comprehensively evaluated the prognostic significance of LVI in PCa. Our findings reveal that LVI is associated with a nearly twofold higher risk of BCR, underscoring its potential role as a critical prognostic marker. In addition, this is the first study to establish a significant association between LVI and both distant metastases and PNI in a meta-analytical setting, further highlighting the aggressive nature of LVI-positive PCa. These results suggest that incorporation of LVI status into clinical decision-making could enhance risk stratification. Randomised studies are needed to validate these associations and investigate the underlying mechanisms.

  ***Author contributions:*** Bartosz Małkiewicz had full access to all the data in the study and takes responsibility for the integrity of the data and the accuracy of the data analysis.

  *Study concept and design*: Karwacki, Szydełko, Małkiewicz.

*Acquisition of data*: Karwacki, Stodolak, Dłubak, Kowalczyk, Holdun.

*Analysis and interpretation of data*: Karwacki, Małkiewicz, Hałoń, Kiełb, Holdun.

*Drafting of the manuscript*: Karwacki, Stodolak, Nowak, Gurwin, Szlasa, Kowalczyk.

*Critical revision of the manuscript for important intellectual content*: Krajewski, Hałoń, Kiełb, Szydełko, Małkiewicz.

*Statistical analysis*: Karwacki, Nowak, Gurwin, Karwacka.

*Obtaining funding*: Małkiewicz, Szydełko.

*Administrative, technical, or material support*: Krajewski, Szydełko, Szlasa, Karwacka.

*Supervision*: Małkiewicz.

*Other*: None.

  ***Financial disclosures:*** Bartosz Małkiewicz certifies that all conflicts of interest, including specific financial interests and relationships and affiliations relevant to the subject matter or materials discussed in the manuscript (eg, employment/affiliation, grants or funding, consultancies, honoraria, stock ownership or options, expert testimony, royalties, or patents filed, received, or pending), are the following: None.

  ***Funding/Support and role of the sponsor:*** This work was supported by grant SUBZ.C090.24.089 from Wroclaw Medical University. The sponsor played no direct role in the study.

  ***Data sharing statement:*** The data supporting the findings of this systematic review and meta-analysis are available on request from the corresponding author.

## References

[1] Sung H., Ferlay J., Siegel R.L. (2021). Global cancer statistics 2020: GLOBOCAN estimates of incidence and mortality worldwide for 36 cancers in 185 countries. CA Cancer J Clin.

[2] Shariat S.F., Khoddami S.M., Saboorian H. (2004). Lymphovascular invasion is a pathological feature of biologically aggressive disease in patients treated with radical prostatectomy. J Urol.

[3] Rakic N., Jamil M., Keeley J. (2021). Evaluation of lymphovascular invasion as a prognostic predictor of overall survival after radical prostatectomy. Urol Oncol.

[4] Karwacki J., Gurwin A., Jaworski A. (2024). Association of lymphovascular invasion with lymph node metastases in prostate cancer—lateralization concept. Cancers.

[5] Loeb S., Roehl K.A., Yu X. (2006). Lymphovascular invasion in radical prostatectomy specimens: prediction of adverse pathologic features and biochemical progression. Urology.

[6] Kozal S., Peyronnet B., Cattarino S. (2015). Influence of pathological factors on oncological outcomes after robot-assisted radical prostatectomy for localized prostate cancer: results of a prospective study. Urol Oncol.

[7] Ng J., Mahmud A., Bass B., Brundage M. (2012). Prognostic significance of lymphovascular invasion in radical prostatectomy specimens. BJU Int.

[8] Huang Y., Huang H., Pan X.W. (2016). The prognostic value of lymphovascular invasion in radical prostatectomy: a systematic review and meta-analysis. Asian J Androl.

[9] Jiang W., Zhang L., Wu B. (2018). The impact of lymphovascular invasion in patients with prostate cancer following radical prostatectomy and its association with their clinicopathological features: an updated PRISMA-compliant systematic review and meta-analysis. Medicine.

[10] Liu H., Zhou H., Yan L. (2017). Prognostic significance of six clinicopathological features for biochemical recurrence after radical prostatectomy: a systematic review and meta-analysis. Oncotarget.

[11] Karwacki J., Stodolak M., Nowak Ł. (2024). Preoperative factors for lymphovascular invasion in prostate cancer: a systematic review and meta-analysis. Int J Mol Sci.

[12] Mottet N., Cornford P., van den Bergh R.C.N. (2023).

[13] van Leenders G.J.L.H., van der Kwast T.H., Grignon D.J. (2020). The 2019 International Society of Urological Pathology (ISUP) consensus conference on grading of prostatic carcinoma. Am J Surg Pathol.

[14] Magi-Galluzzi C., Evans A.J., Delahunt B. (2011). International Society of Urological Pathology (ISUP) consensus conference on handling and staging of radical prostatectomy specimens. Working group 3: extraprostatic extension, lymphovascular invasion and locally advanced disease. Mod Pathol.

[15] Page M.J., McKenzie J.E., Bossuyt P.M. (2021). The PRISMA 2020 statement: an updated guideline for reporting systematic reviews. Rev Esp Cardiol.

[16] Higgins J., Thomas J., Chandler J. (2023). Cochrane handbook for systematic reviews of interventions version 6.4.

[17] McGuinness L.A., Higgins J.P.T. (2021). Risk-of-bias VISualization (robvis): an R package and Shiny web app for visualizing risk-of-bias assessments. Res Synth Methods.

[18] Al Qa’qa’ S., Downes M.R., Jain R., van der Kwast T. (2023). Morphologic pattern, frequency, and spatial distribution of lymphovascular invasion foci in radical prostatectomy specimens. Int J Surg Pathol.

[19] Andersen S., Richardsen E., Nordby Y. (2014). Disease-specific outcomes of radical prostatectomies in Northern Norway; a case for the impact of perineural infiltration and postoperative PSA-doubling time. BMC Urol.

[20] Andras I., Crisan N., Coman R.T. (2016). Oncological results at 2 years after robotic radical prostatectomy – the Romanian experience. Cent Eur J Urol.

[21] Antunes A.A., Srougi M., Dall’oglio M.F. (2006). Microvascular invasion is an independent prognostic factor in patients with prostate cancer treated with radical prostatectomy. Int Braz J Urol.

[22] Ariafar A., Zeighami S., Salehipour M., Ahmed F., Shahabi Z., Nikbakht H.A. (2021). An investigation of the pathology report of prostate cancer patients with radical prostatectomy in Southern Iran: a cross-sectional study. Middle East J Cancer.

[23] Babaian R.J., Troncoso P., Bhadkamkar V.A., Johnston D.A. (2001). Analysis of clinicopathologic factors predicting outcome after radical prostatectomy. Cancer.

[24] Bargão Santos P., Lobo J., Félix A. (2020). The inflammation-related biomarker CXCR7 independently predicts patient outcome after radical prostatectomy. Urol Oncol.

[25] Baydar D.E., Baseskioglu B., Ozen H., Geyik P.O. (2008). Prognostic significance of lymphovascular invasion in clinically localized prostate cancer after radical prostatectomy. Sci World J.

[26] Brooks J.P., Albert P.S., Wilder R.B., Gant D.A., McLeod D.G., Poggi M.M. (2005). Long-term salvage radiotherapy outcome after radical prostatectomy and relapse predictors. J Urol.

[27] Brooks J.P., Albert P.S., O’Connell J., McLeod D.G., Poggi M.M. (2006). Lymphovascular invasion in prostate cancer: prognostic significance in patients treated with radiotherapy after radical prostatectomy. Cancer.

[28] Celik S., Eker A., Bozkurt İ.H. (2020). Factors affecting biochemical recurrence of prostate cancer after radical prostatectomy in patients with positive and negative surgical margin. Prostate Int.

[29] Chen Z., Pham H., Abreu A. (2021). Prognostic value of cribriform size, percentage, and intraductal carcinoma in Gleason score 7 prostate cancer with cribriform Gleason pattern 4. Hum Pathol.

[30] Cheng L., Jones T.D., Lin H. (2005). Lymphovascular invasion is an independent prognostic factor in prostatic adenocarcinoma. J Urol.

[31] Cho I.C., Chung H.S., Cho K.S. (2010). Bcl-2 as a predictive factor for biochemical recurrence after radical prostatectomy: an interim analysis. Cancer Res Treat.

[32] Chromecki T.F., Cha E.K., Pummer K. (2012). Prognostic value of insulin-like growth factor II mRNA binding protein 3 in patients treated with radical prostatectomy. BJU Int.

[33] Chung D.Y., Koh D.H., Goh H.J. (2018). Clinical significance and predictors of oncologic outcome after radical prostatectomy for invisible prostate cancer on multiparametric MRI. BMC Cancer.

[34] de la Taille A., Rubin M.A., Buttyan R. (2000). Is microvascular invasion on radical prostatectomy specimens a useful predictor of PSA recurrence for prostate cancer patients?. Eur Urol.

[35] Dere Y., Altinboga A.A., Bal K., Calli A., Ermete M., Sari A.A. (2017). The histopathological parameters affecting biochemical recurrence in radical prostatectomies. J Coll Phys Surg Pak.

[36] Epstein J.I., Partin A.W., Potter S.R., Walsh P.C. (2000). Adenocarcinoma of the prostate invading the seminal vesicle: prognostic stratification based on pathologic parameters. Urology.

[37] Fajkovic H., Mathieu R., Lucca I. (2016). Validation of lymphovascular invasion is an independent prognostic factor for biochemical recurrence after radical prostatectomy. Urol Oncol.

[38] Ferrari M.K., McNeal J.E., Malhotra S.M., Brooks J.D. (2004). Vascular invasion predicts recurrence after radical prostatectomy: stratification of risk based on pathologic variables. Urology.

[39] Fujimura T., Fukuhara H., Taguchi S. (2017). Robot-assisted radical prostatectomy significantly reduced biochemical recurrence compared to retro pubic radical prostatectomy. BMC Cancer.

[40] Furukawa J., Miyake H., Inoue T.A., Ogawa T., Tanaka H., Fujisawa M. (2016). Oncologic outcome of radical prostatectomy as monotherapy for men with high-risk prostate cancer. Curr Urol.

[41] Galiabovitch E., Hovens C.M., Peters J.S. (2017). Routinely reported ‘equivocal’ lymphovascular invasion in prostatectomy specimens is associated with adverse outcomes. BJU Int.

[42] Gesztes W., Schafer C., Young D. (2022). Focal p53 protein expression and lymphovascular invasion in primary prostate tumors predict metastatic progression. Sci Rep.

[43] Goenka A., Magsanoc J.M., Pei X. (2012). Long-term outcomes after high-dose postprostatectomy salvage radiation treatment. Int J Radiat Oncol Biol Phys.

[44] Gottlieb J., Chang S.C., Choe J. (2023). Characterization of lymph node tumor burden in node-positive prostate cancer patients after robotic-assisted radical prostatectomy with extended pelvic lymph node dissection. Cancers.

[45] Gun E., Ocal I. (2021). Cribriform glands are associated with worse outcome than other pattern 4 subtypes: a study of prognostic and clinicopathological characteristics of prostate adenocarcinoma with an emphasis on grade groups. Int J Clin Pract.

[46] Hashimoto T., Nakashima J., Kashima T. (2020). Predicting factors for progression to castration resistance prostate cancer after biochemical recurrence in patients with clinically localized prostate cancer who underwent radical prostatectomy. Int J Clin Oncol.

[47] Hashimoto T., Yoshioka K., Nagao G. (2015). Prediction of biochemical recurrence after robot-assisted radical prostatectomy: analysis of 784 Japanese patients. Int J Urol.

[48] Herman C.M., Wilcox G.E., Kattan M.W., Scardino P.T., Wheeler T.M. (2000). Lymphovascular invasion as a predictor of disease progression in prostate cancer. Am J Surg Pathol.

[49] Hong J., Kwon Y., Kim I. (2017). Risk stratification for disease progression in pT3 prostate cancer after robot-assisted radical prostatectomy. Asian J Androl.

[50] Hsieh C.Y., Lin C.Y., Wang S.S. (2023). Impact of clinicopathological characteristics and tissue inhibitor of metalloproteinase-3 polymorphism Rs9619311 on biochemical recurrence in Taiwanese patients with prostate cancer. Int J Environ Res Public Health.

[51] Huang S.P., Huang C.Y., Wang J.S. (2007). Prognostic significance of p53 and X-ray repair cross-complementing group 1 polymorphisms on prostate-specific antigen recurrence in prostate cancer post–radical prostatectomy. Clin Cancer Res.

[52] Ito K., Nakashima J., Mukai M. (2003). Prognostic implication of microvascular invasion in biochemical failure in patients treated with radical prostatectomy. Urol Int.

[53] Jamil M., Rakic N., Sood A. (2021). Impact of lymphovascular invasion on overall survival in patients with prostate cancer following radical prostatectomy: stage-per-stage analysis. Clin Genitourin Cancer.

[54] Jeon H.G., Bae J., Yi J.S., Hwang I.S., Lee S.E., Lee E. (2009). Perineural invasion is a prognostic factor for biochemical failure after radical prostatectomy. International Journal of Urology.

[55] Jeong S.U., Kekatpure A.K., Park J.M. (2017). Diverse immunoprofile of ductal adenocarcinoma of the prostate with an emphasis on the prognostic factors. J Pathol Transl Med.

[56] Jeong J.U., Nam T.K., Song J.Y. (2019). Prognostic significance of lymphovascular invasion in patients with prostate cancer treated with postoperative radiotherapy. Radiat Oncol J.

[57] Joung J.Y., Yang S.O., Jeong I.G. (2007). Reverse transcriptase-polymerase chain reaction and immunohistochemical studies for detection of prostate stem cell antigen expression in prostate cancer: potential value in molecular staging of prostate cancer. Int J Urol.

[58] Jung J.H., Lee J.W., Arkoncel F.R.P. (2011). Significance of perineural invasion, lymphovascular invasion, and high-grade prostatic intraepithelial neoplasia in robot-assisted laparoscopic radical prostatectomy. Ann Surg Oncol.

[59] Kamitani R., Matsumoto K., Kosaka T. (2021). Evaluation of Gleason grade group 5 in a contemporary prostate cancer grading system and literature review. Clin Genitourin Cancer.

[60] Kang Y.J., Kim H.S., Jang W.S. (2017). Impact of lymphovascular invasion on lymph node metastasis for patients undergoing radical prostatectomy with negative resection margin. BMC Cancer.

[61] Kang M., Oh J.J., Lee S., Hong S.K., Lee S.E., Byun S.S. (2016). Perineural invasion and lymphovascular invasion are associated with increased risk of biochemical recurrence in patients undergoing radical prostatectomy. Ann Surg Oncol.

[62] Kawase M., Ebara S., Tatenuma T. (2024). Prognostic importance of lymphovascular invasion for specific subgroup of patients with prostate cancer after robot-assisted radical prostatectomy (the MSUG94 group). Ann Surg Oncol.

[63] Kim S.J., Park M.U., Chae H.K. (2022). Overweight and obesity as risk factors for biochemical recurrence of prostate cancer after radical prostatectomy. Int J Clin Oncol.

[64] Kim S.H., Park W.S., Lee S.J. (2015). The quantified level of circulating prostate stem cell antigen mRNA relative to GAPDH level is a clinically significant indictor for predicting biochemical recurrence in prostate cancer patients after radical prostatectomy. Biomed Res Int.

[65] Kneebone A., Hruby G., Harris G. (2018). Contemporary salvage post prostatectomy radiotherapy: early implementation improves biochemical control. J Med Imaging Radiat Oncol.

[66] Koparal M.Y., Sözen T.S., Aslan G. (2021). Prognostic significance of surgical margin status and Gleason grade at the positive surgical margin in predicting biochemical recurrence after radical prostatectomy in a Turkish patient cohort. Bull Urooncol.

[67] Lee J.T., Lee S., Yun C.J. (2010). Prediction of perineural invasion and its prognostic value in patients with prostate cancer. Korean J Urol.

[68] Leng Y.H., Lee W.J., Yang S.O., Lee J.K., Jung T.Y., Kim Y.B. (2013). Oncologic outcomes of patients with Gleason score 7 and tertiary Gleason pattern 5 after radical prostatectomy. Korean J Urol.

[69] Liauw S.L., Webster W.S., Pistenmaa D.A., Roehrborn C.G. (2003). Salvage radiotherapy for biochemical failure of radical prostatectomy: a single-institution experience. Urology.

[70] Luo H.L., Chiang P.H., Chen Y.T., Cheng Y.T. (2012). Lymphovascular invasion is a pathological feature related to aggressive cancer behavior and predicts early recurrence in prostate cancer. Kaohsiung J Med Sci.

[71] May M., Kaufmann O., Hammermann F., Loy V., Siegsmund M. (2007). Prognostic impact of lymphovascular invasion in radical prostatectomy specimens. BJU Int.

[72] Mian B.M., Troncoso P., Okihara K. (2002). Outcome of patients with Gleason score 8 or higher prostate cancer following radical prostatectomy alone. J Urol.

[73] Micoogullari U., Cakici M.C., Kisa E. (2021). A risk grouping algorithm for predicting factors of persistently elevated prostate-specific antigen in patients following robot-assisted radical prostatectomy. Int J Clin Pract.

[74] Mitsuzuka K., Narita S., Koie T. (2015). Lymphovascular invasion is significantly associated with biochemical relapse after radical prostatectomy even in patients with pT2N0 negative resection margin. Prostate Cancer Prostat Dis.

[75] Miyai K., Divatia M.K., Shen S.S., Miles B.J., Ayala A.G., Ro J.Y. (2014). Clinicopathological analysis of intraductal proliferative lesions of prostate: Intraductal carcinoma of prostate, high-grade prostatic intraepithelial neoplasia, and atypical cribriform lesion. Hum Pathol.

[76] Mizuno R., Nakashima J., Mukai M. (2006). Maximum tumor diameter is a simple and valuable index associated with the local extent of disease in clinically localized prostate cancer. Int J Urol.

[77] Mizuno R., Nakashima J., Mukai M. (2009). Tumour length of the largest focus predicts prostate-specific antigen-based recurrence after radical prostatectomy in clinically localized prostate cancer. BJU Int.

[78] Numbere N., Teramoto Y., Gurung P.M.S., Goto T., Yang Z., Miyamoto H. (2022). The clinical impact of pT3a lesions in patients with pT3b prostate cancer undergoing radical prostatectomy a proposal for a new pT3b subclassification. Arch Pathol Lab Med.

[79] Ohno Y., Ohori M., Nakashima J. (2016). Association between preoperative serum total cholesterol level and biochemical recurrence in prostate cancer patients who underwent radical prostatectomy. Mol Clin Oncol.

[80] Oufattole J., Dey T., D’Amico A.V., van Leenders G.J.L.H., Acosta A.M. (2023). Cribriform morphology is associated with higher risk of biochemical recurrence after radical prostatectomy in patients with Grade Group 5 prostate cancer. Histopathology.

[81] Özkanli S.Ş., Zemheri I.E., Yildirim A. (2014). Gleason score at the margin can predict biochemical recurrence after radical prostatectomy, in addition to preoperative PSA and surgical margin status. Turk J Med Sci.

[82] Özsoy M., D’Andrea D., Moschini M. (2018). Tertiary Gleason pattern in radical prostatectomy specimens is associated with worse outcomes than the next higher Gleason score group in localized prostate cancer. Urol Oncol.

[83] Pagano M.J., Whalen M.J., Paulucci D.J. (2016). Predictors of biochemical recurrence in pT3b prostate cancer after radical prostatectomy without adjuvant radiotherapy. Prostate.

[84] Park Y.H., Kim Y., Yu H. (2016). Is lymphovascular invasion a powerful predictor for biochemical recurrence in pT3 N0 prostate cancer? Results from the K-CaP database. Sci Rep.

[85] Psutka S.P., Feldman A.S., Rodin D., Olumi A.F., Wu C.L., McDougal W.S. (2011). Men with organ-confined prostate cancer and positive surgical margins develop biochemical failure at a similar rate to men with extracapsular extension. Urology.

[86] Quinn D.I., Henshall S.M., Haynes A.M. (2001). Prognostic significance of pathologic features in localized prostate cancer treated with radical prostatectomy: implications for staging systems and predictive models. J Clin Oncol.

[87] Rodrigues I., Ferreira C., Gonçalves J. (2021). Pathological stage, surgical margin and lymphovascular invasion as prognostic factors after salvage radiotherapy for post-prostatectomy relapsed prostate cancer — outcomes and optimization strategies. Rep Pract Oncol Radiother.

[88] Safdieh J.J., Schwartz D., Weiner J. (2014). Long-term tolerance and outcomes for dose escalation in early salvage post-prostatectomy radiation therapy. Radiat Oncol J.

[89] Salomao D.R., Graham S.D., Bostwick D.G. (1995). Microvascular invasion in prostate cancer correlates with pathologic stage. Arch Pathol Lab Med.

[90] Sathianathen N.J., Furrer M.A., Mulholland C.J. (2023). Lymphovascular invasion at the time of radical prostatectomy adversely impacts oncological outcomes. Cancers.

[91] Sertkaya Z., Öztürk M.İ., Koca O., Güneş M., Karaman M.İ. (2014). Predictive values for extracapsular extension in prostate cancer patients with PSA values below 10 ng/mL. Turk Uroloji Dergisi.

[92] Sevcenco S., Mathieu R., Baltzer P. (2016). The prognostic role of preoperative serum C-reactive protein in predicting the biochemical recurrence in patients treated with radical prostatectomy. Prostate Cancer Prostat Dis.

[93] Shin T.J., Jung W., Ha J.Y., Kim B.H., Kim Y.H. (2021). The significance of the visible tumor on preoperative magnetic resonance imaging in localized prostate cancer. Prostate Int.

[94] Stamey T.A., Yemoto C.M., McNeal J.E., Sigal B.M., Johnstone I.M. (2000). Prostate cancer is highly predictable: a prognostic equation based on all morphological variables in radical prostatectomy specimens. J Urol.

[95] Taguchi S., Shiraishi K., Fukuhara H. (2016). Impact of Gleason pattern 5 including tertiary pattern 5 on outcomes of salvage treatment for biochemical recurrence in pT2–3N0M0 prostate cancer. Int J Clin Oncol.

[96] Taverna G., Grizzi F., Colombo P. (2015). Two-dimensional neovascular complexity is significantly higher in nontumor prostate tissue than in low-risk prostate cancer. Korean J Urol.

[97] Tokuda Y., Carlino L.J., Gopalan A. (2010). Prostate cancer topography and patterns of lymph node metastasis. Am J Surg Pathol.

[98] van den Ouden D., Kranse R., Hop W.C.J., van der Kwast T.H., Schröder F.H. (1998). Microvascular invasion in prostate cancer: prognostic significance in patients treated by radical prostatectomy for clinically localized carcinoma. Urol Int.

[99] Vau N., Henriques V., Cheng L. (2019). Predicting biochemical recurrence after radical prostatectomy: the role of prognostic grade group and index tumor nodule. Hum Pathol.

[100] Wessels F., Schmitt M., Krieghoff-Henning E. (2021). Deep learning approach to predict lymph node metastasis directly from primary tumour histology in prostate cancer. BJU Int.

[101] Whittemore D.E., Hick E.J., Carter M.R., Moul J.W., Miranda-Sousa A.J., Sexton W.J. (2008). Significance of tertiary Gleason pattern 5 in Gleason score 7 radical prostatectomy specimens. J Urol.

[102] Yamamoto S., Kawakami S., Yonese J. (2008). Lymphovascular invasion is an independent predictor of prostate-specific antigen failure after radical prostatectomy in patients with pT3aN0 prostate cancer. Int J Urol.

[103] Yee D.S., Shariat S.F., Lowrance W.T. (2011). Prognostic significance of lymphovascular invasion in radical prostatectomy specimens. BJU Int.

[104] Yoneda K., Utsumi T., Somoto T. (2018). External validation of two web-based postoperative nomograms predicting the probability of early biochemical recurrence after radical prostatectomy: a retrospective cohort study. Jpn J Clin Oncol.

[105] You D., Jeong I.G., Song C. (2014). High percent tumor volume predicts biochemical recurrence after radical prostatectomy in pathological stageT3a prostate cancer with a negative surgical margin. Int J Urol.

[106] Yuksel M., Karamik K., Anil H., Islamoglu E., Ates M., Savas M. (2017). Factors affecting surgical margin positivity in robotic assisted radical prostatectomy. Arch Ital Urol Androl.

[107] Karwacki J., Łątkowska M., Jarocki M. (2024). The clinical meaning of lymphovascular invasion: preoperative predictors and postoperative implications in prostate cancer – a retrospective study. Front Oncol.

[108] Grignon D.J. (2018). Prostate cancer reporting and staging: needle biopsy and radical prostatectomy specimens. Mod Pathol.

