# Supplementary material for: Association of Lymphovascular Invasion with Biochemical Recurrence and Adverse Pathological Characteristics of Prostate Cancer: A Systematic Review and Meta-analysis
Source: Eur Urol Open Sci. 2024 Oct 8;69:112–26. doi: 10.1016/j.euros.2024.09.007 (PMC11490882; doi:10.1016/j.euros.2024.09.007)
Supplement: Supplementary Data 1 [file mmc1.docx]

**Association of lymphovascular invasion with biochemical recurrence and adverse pathological characteristics in prostate cancer: a systematic review and meta-analysis**

**Supplementary material**

- **Supplementary Figure 1.** Risk of bias analysis – part 1.
- **Supplementary Figure 2.** Risk of bias analysis – part 2.
- **Supplementary Figure 3.** Forest plot of HRs in RE model predicting BCR according to LVI in subgroups: multivariate and univariate analyses.
- **Supplementary Figure 4.** Forest plot of HRs in RE model predicting BCR according to LVI in subgroups: prospective and retrospective studies.
- **Supplementary Figure 5.** Forest plot of HRs in RE model predicting BCR according to LVI in subgroups: single center and multi center/registry based studies.
- **Supplementary Figure 6.** Forest plot of HRs in RE model predicting BCR according to LVI in subgroups: publication date before 2016 and in/after 2016.
- **Supplementary Figure 7.** Forest plot of HRs in RE model predicting BCR according to LVI in subgroups: sample size of less than 500 patients and more than or equal to 500 patients.
- **Supplementary Figure 8.** Forest plot of HRs in RE model predicting BCR according to LVI in subgroups: median/mean follow-up of less than 30 months and more than or equal to 30 months.
- **Supplementary Figure 9.** Forest plot of HRs in RE model predicting BCR according to LVI in subgroups: median/mean age of less than 65 years and more than or equal to 65 years.
- **Supplementary Figure 10.** Forest plot of HRs in RE model predicting BCR according to LVI in subgroups: median/mean PSA of less than 10 ng/ml and more than or equal to 10 ng/ml.
- **Supplementary Figure 11.** Forest plot of HRs in RE model predicting BCR according to LVI in subgroups: provided and unprovided definitions of LVI.
- **Supplementary Figure 12.** Forest plot of HRs in RE model predicting BCR according to LVI in subgroups: less than 15% and greater than or equal to 15% LVI-positive men in the patient population.
- **Supplementary Figure 13.** Forest plot of HRs in RE model predicting BCR according to LVI in subgroups: PSA cutoff value of >0.1 ng/ml, >0.2 ng/ml, >0.2+nadir ng/ml, or >0.4 ng/ml.
- **Supplementary Figure 14.** Forest plot of ORs in RE model predicting higher pT stage according to LVI.
- **Supplementary Figure 15.** Forest plot of ORs in RE model predicting GS according to LVI.
- **Supplementary Figure 16.** Forest plot of ORs in RE model predicting LNM according to LVI.
- **Supplementary Figure 17.** Forest plot of ORs in FE model predicting distant metastasis according to LVI.
- **Supplementary Figure 18.** Forest plot of ORs in RE model predicting PSM according to LVI.
- **Supplementary Figure 19.** Forest plot of ORs in RE model predicting EPE according to LVI.
- **Supplementary Figure 20.** Forest plot of ORs in RE model predicting SVI according to LVI.
- **Supplementary Figure 21.** Forest plot of ORs in FE model predicting PNI according to LVI.
- **Supplementary Figure 22.** Funnel plot for the evaluation of potential publication bias comprising 51 studies included in the main meta-analysis on BCR with FE model applied.


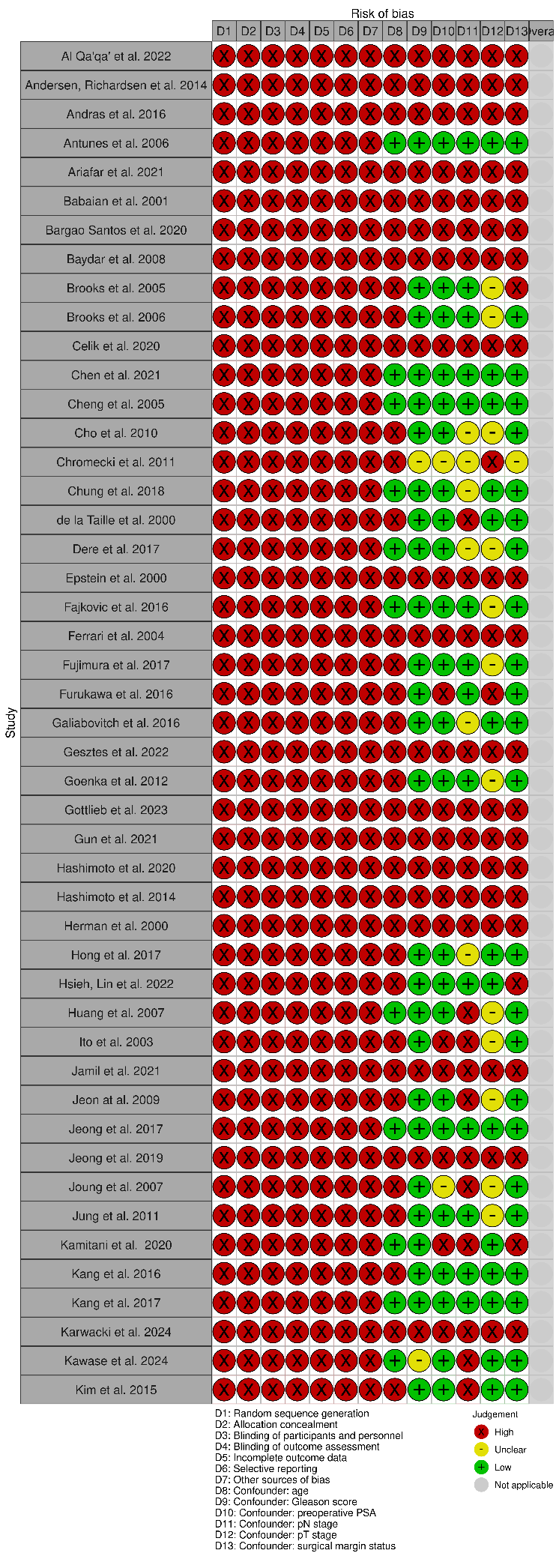


**Supplementary Figure 1**. Risk of bias analysis – part 1.


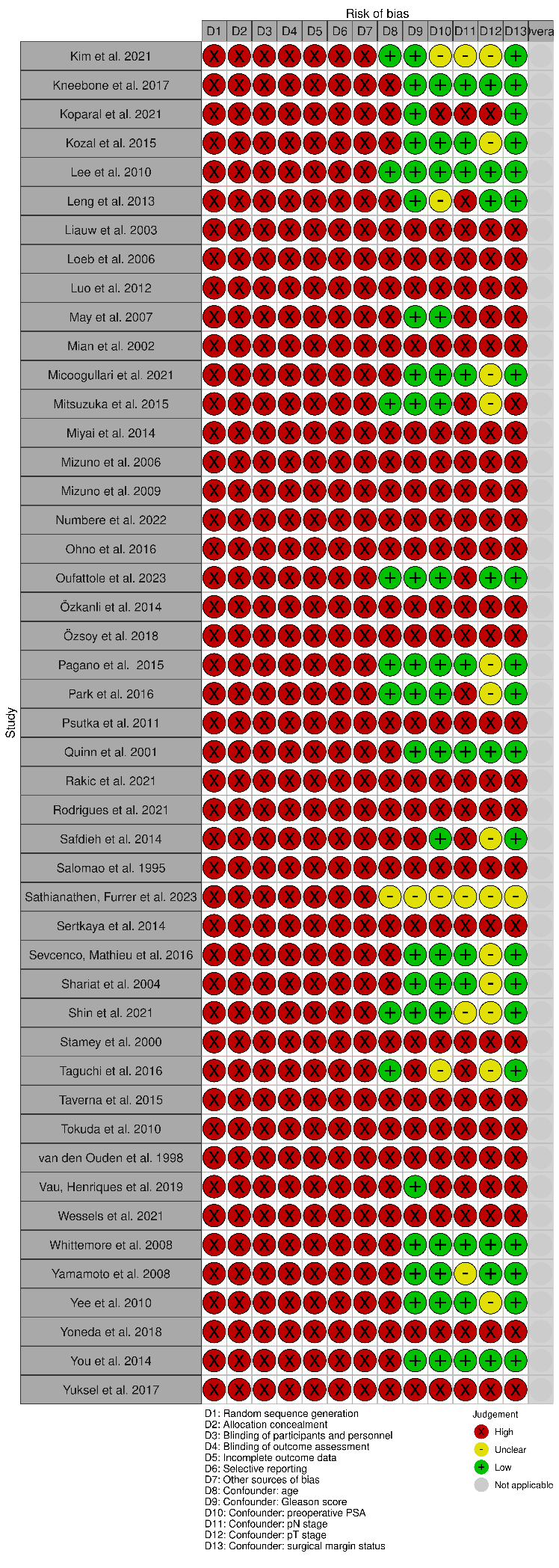


**Supplementary Figure 2**. Risk of bias analysis – part 2.


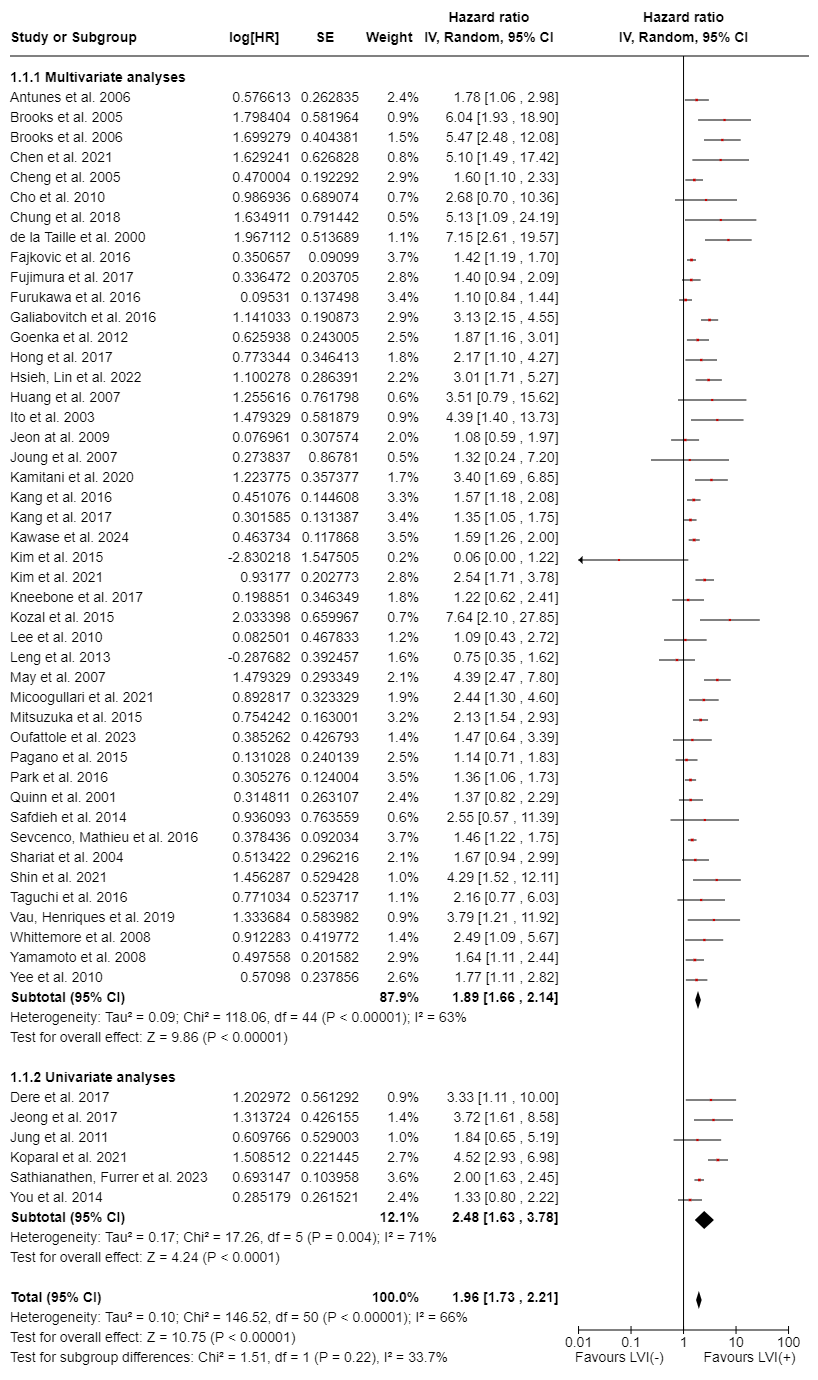


**Supplementary Figure 3.** Forest plot of hazard ratios (HR) in random effects model predicting biochemical recurrence (BCR) according to lymphovascular invasion status in subgroups: multivariate and univariate analysis. CI = confidence interval; df = degree of freedom; M-H = Mantel–Haenszel; SD = standard deviation.


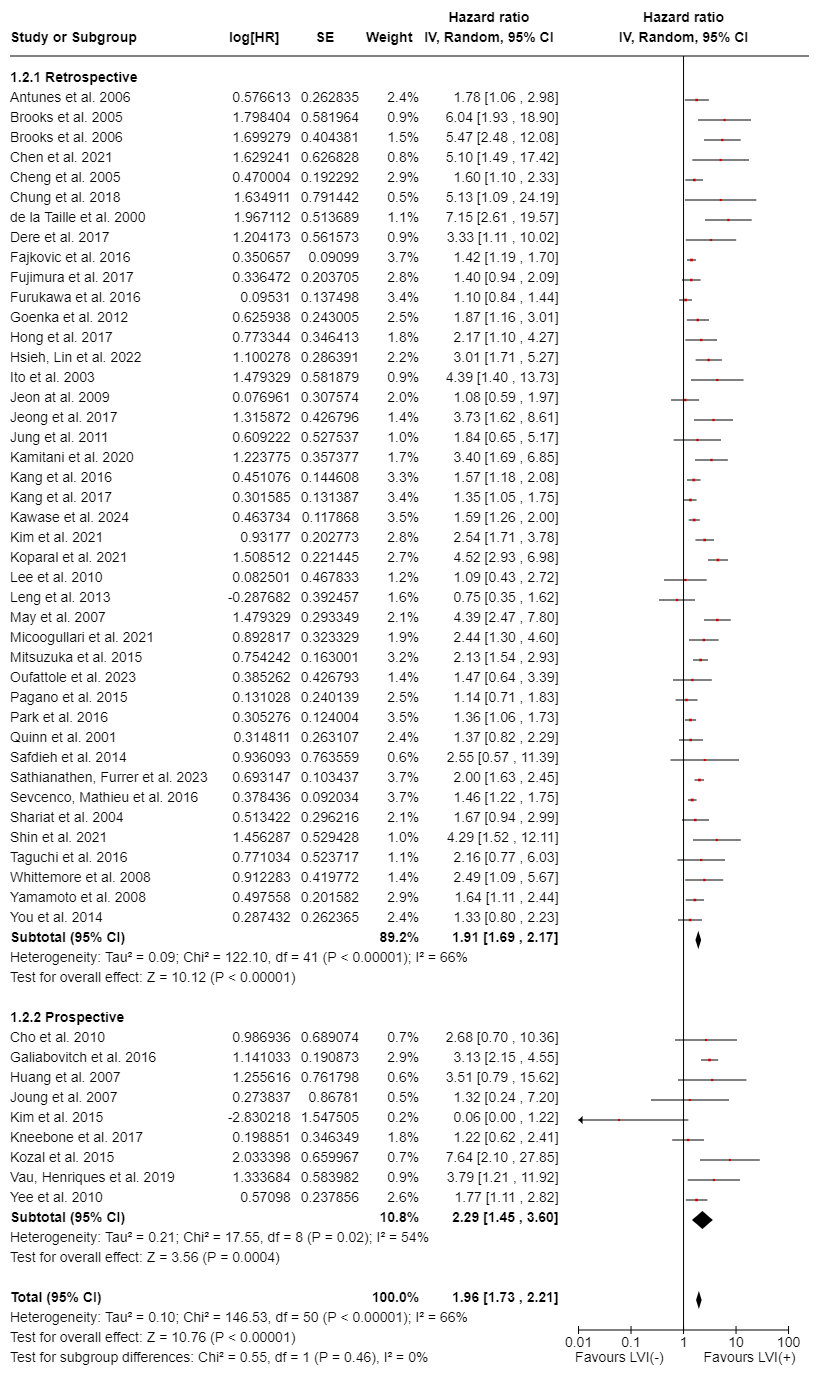


**Supplementary Figure 4.** Forest plot of hazard ratios (HR) in random effects model predicting biochemical recurrence (BCR) according to lymphovascular invasion status in subgroups: prospective and retrospective studies. CI = confidence interval; df = degree of freedom; M-H = Mantel–Haenszel; SD = standard deviation.


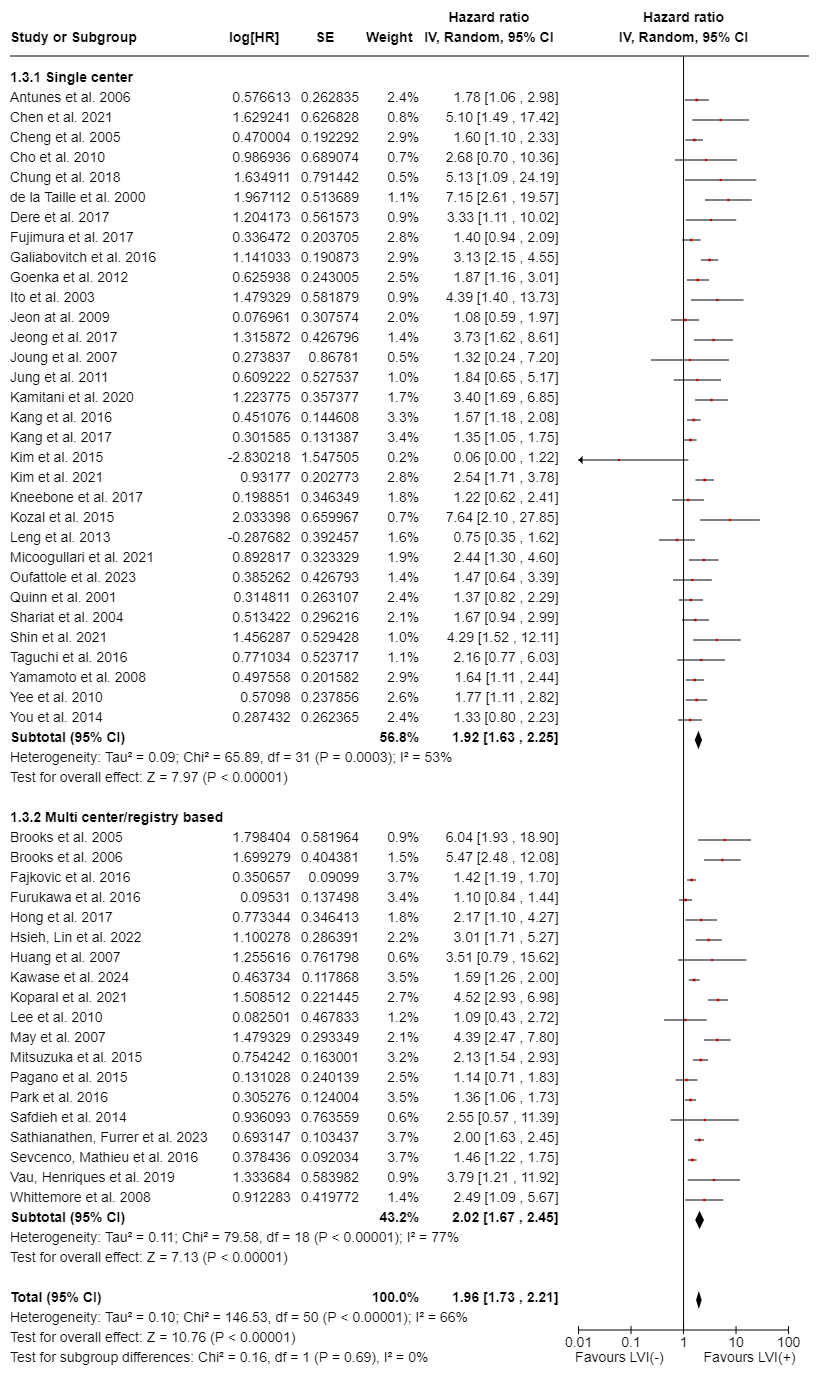


**Supplementary Figure 5.** Forest plot of hazard ratios (HR) in random effects model predicting biochemical recurrence (BCR) according to lymphovascular invasion status in subgroups: single and multi-center studies. CI = confidence interval; df = degree of freedom; M-H = Mantel–Haenszel; SD = standard deviation.


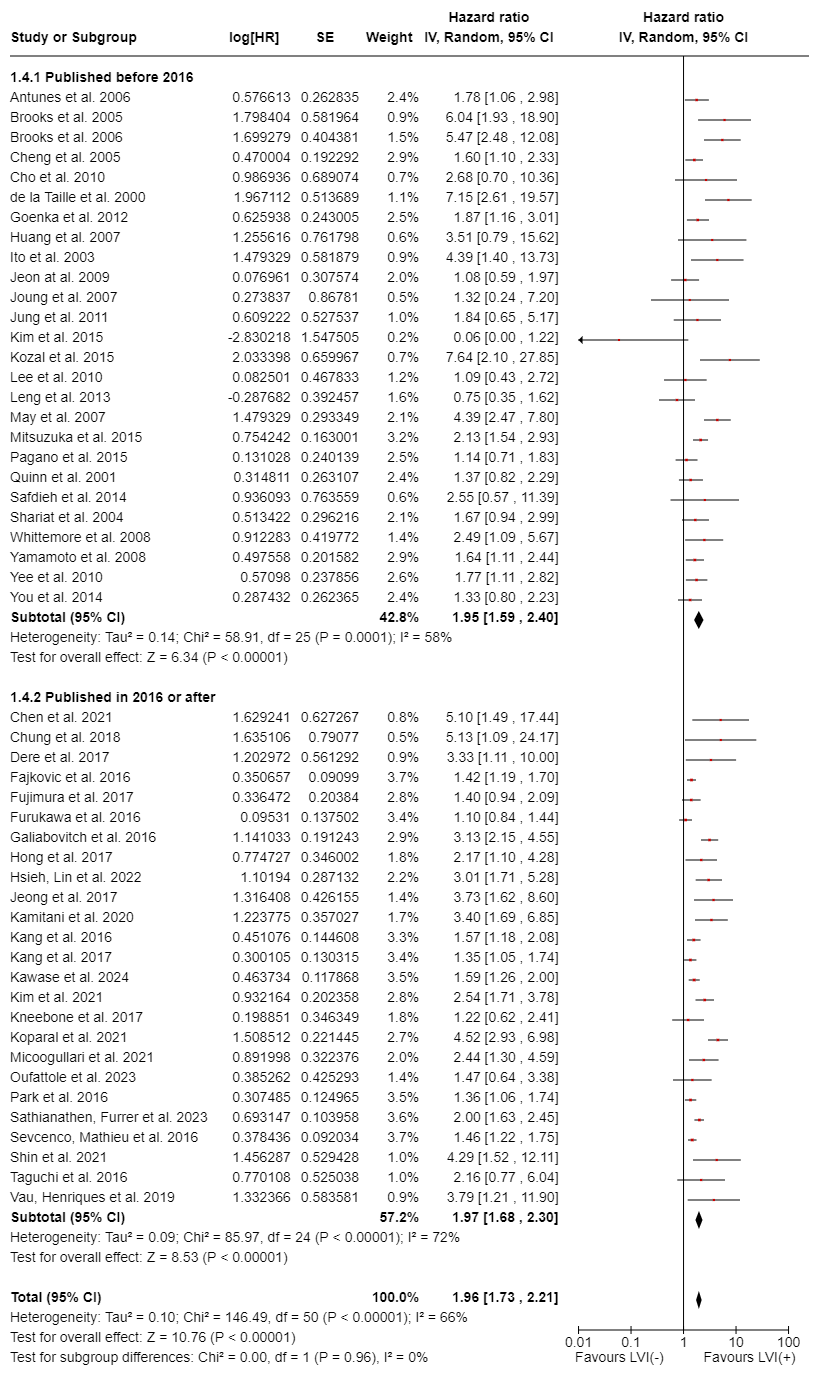


**Supplementary Figure 6.** Forest plot of hazard ratios (HR) in random effects model predicting biochemical recurrence (BCR) according to lymphovascular invasion status in subgroups: date of publication – before 2016 and in of after 2016. CI = confidence interval; df = degree of freedom; M-H = Mantel–Haenszel; SD = standard deviation.


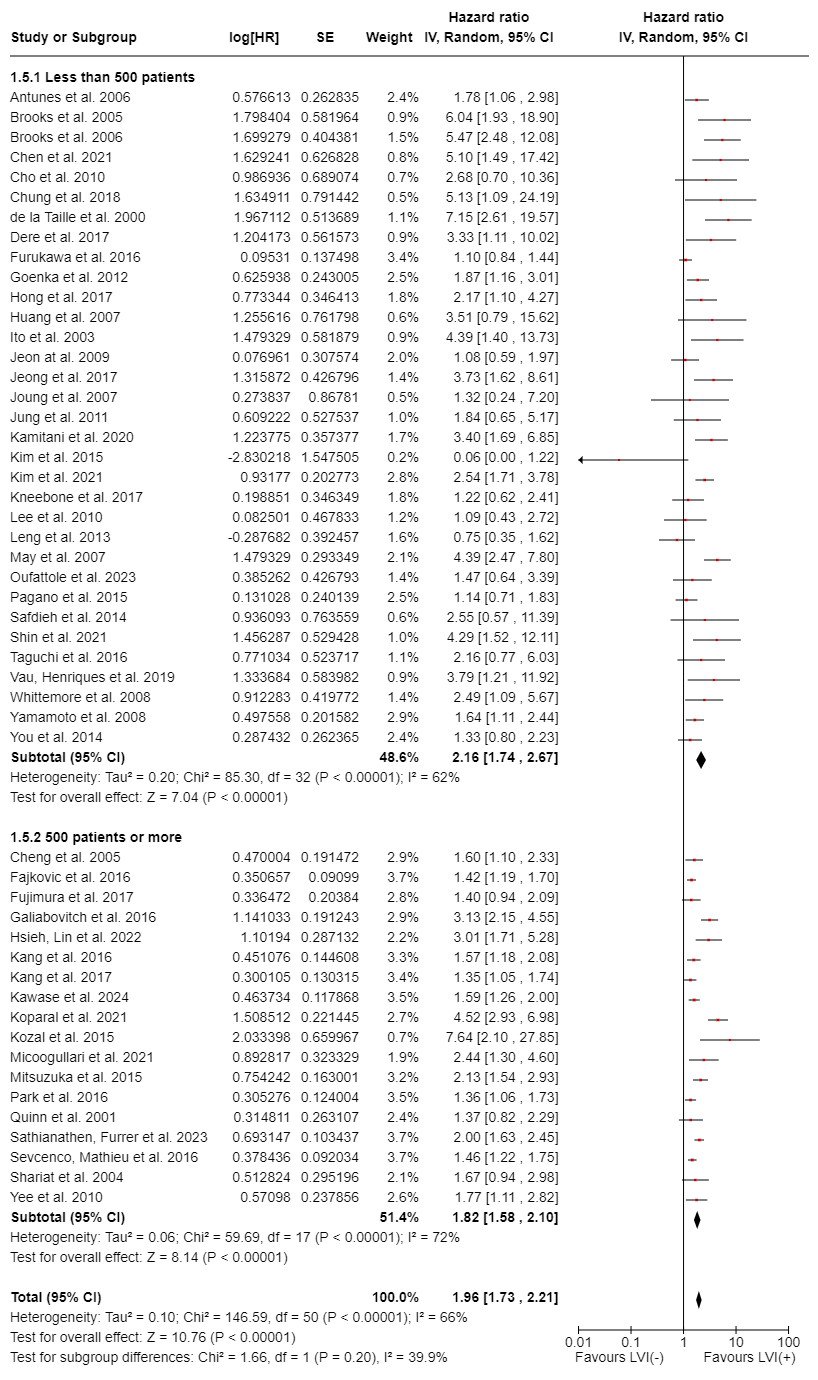


**Supplementary Figure 7.** Forest plot of hazard ratios (HR) in random effects model predicting biochemical recurrence (BCR) according to lymphovascular invasion status in subgroups: number of patients – less than 500 men and greater than or equal to 500 men. CI = confidence interval; df = degree of freedom; M-H = Mantel–Haenszel; SD = standard deviation.


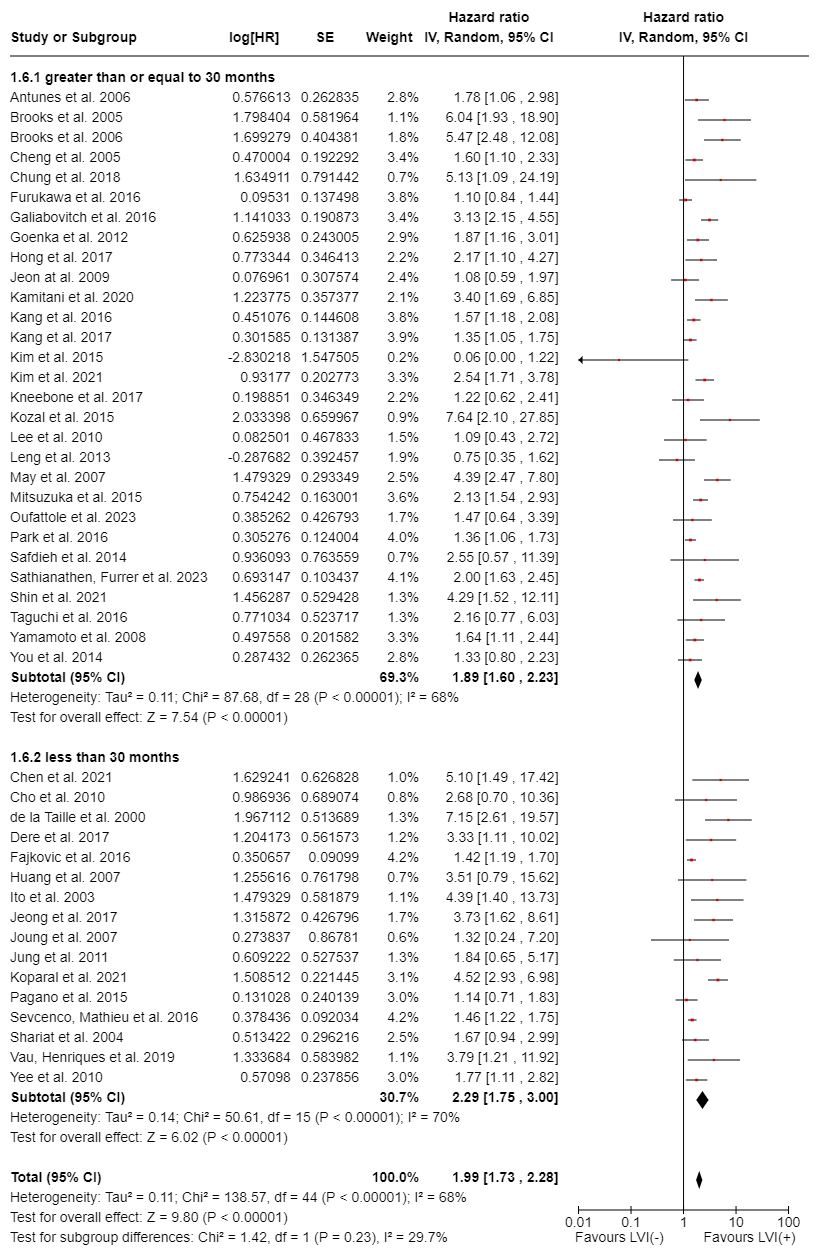


**Supplementary Figure 8.** Forest plot of hazard ratios (HR) in random effects model predicting biochemical recurrence (BCR) according to lymphovascular invasion status in subgroups: follow-up time – less than 30 months and greater than or equal to 30 months. CI = confidence interval; df = degree of freedom; M-H = Mantel–Haenszel; SD = standard deviation.


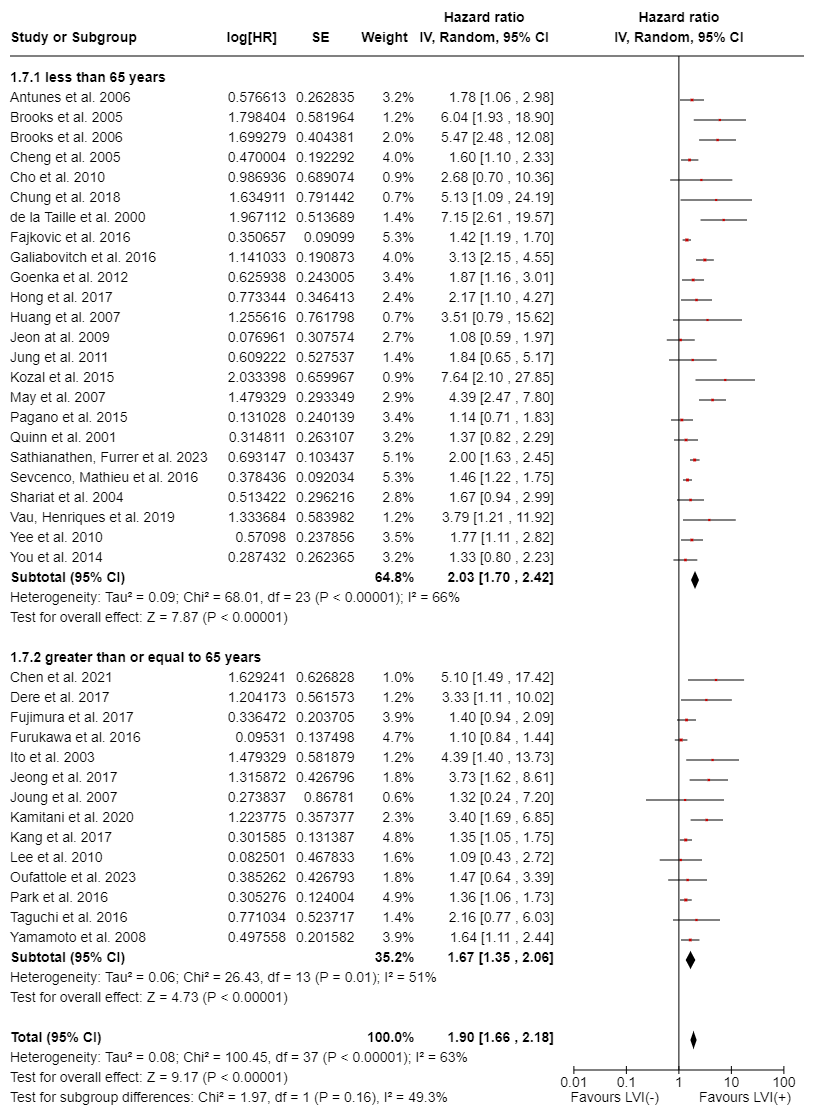


**Supplementary Figure 9.** Forest plot of hazard ratios (HR) in random effects model predicting biochemical recurrence (BCR) according to lymphovascular invasion status in subgroups: age – less than 65 years and greater than or equal to 65 years. CI = confidence interval; df = degree of freedom; M-H = Mantel–Haenszel; SD = standard deviation.


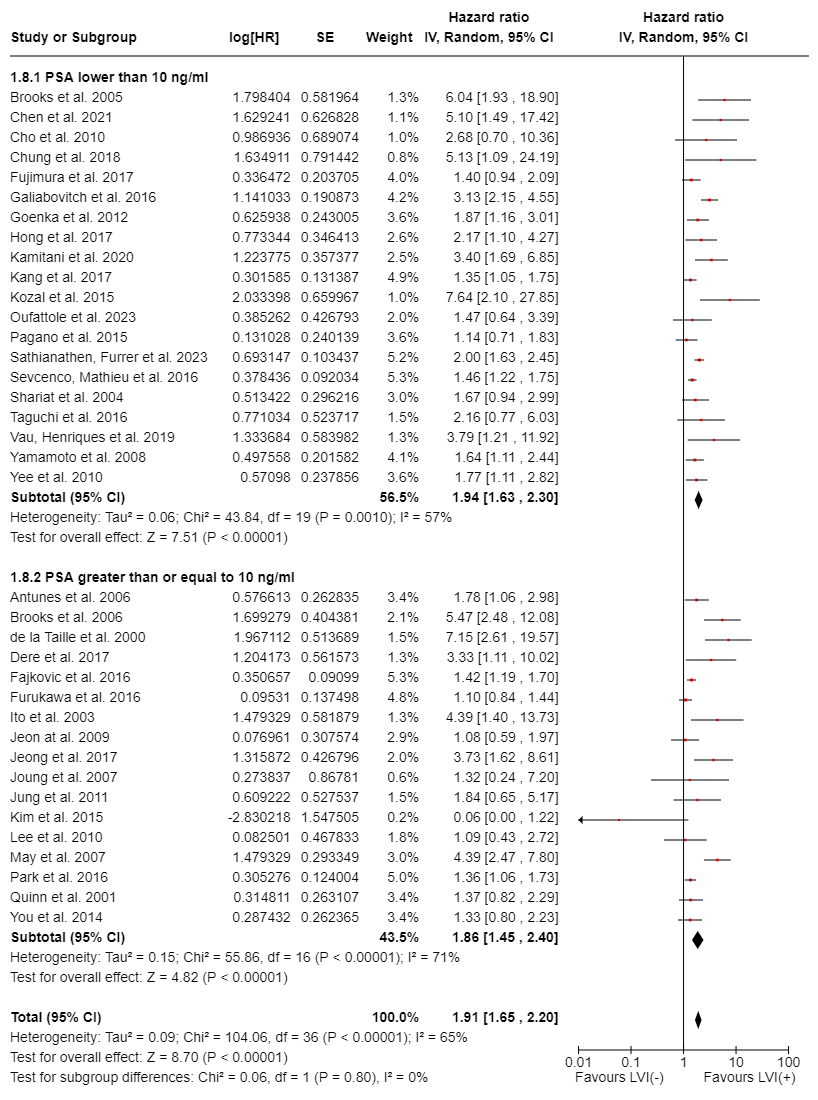


**Supplementary Figure 10.** Forest plot of hazard ratios (HR) in random effects model predicting biochemical recurrence (BCR) according to lymphovascular invasion status in subgroups: prostate specific antigen (PSA) – lower than 10 ng/ml and greater than or equal to 10 ng/ml. CI = confidence interval; df = degree of freedom; M-H = Mantel–Haenszel; SD = standard deviation.


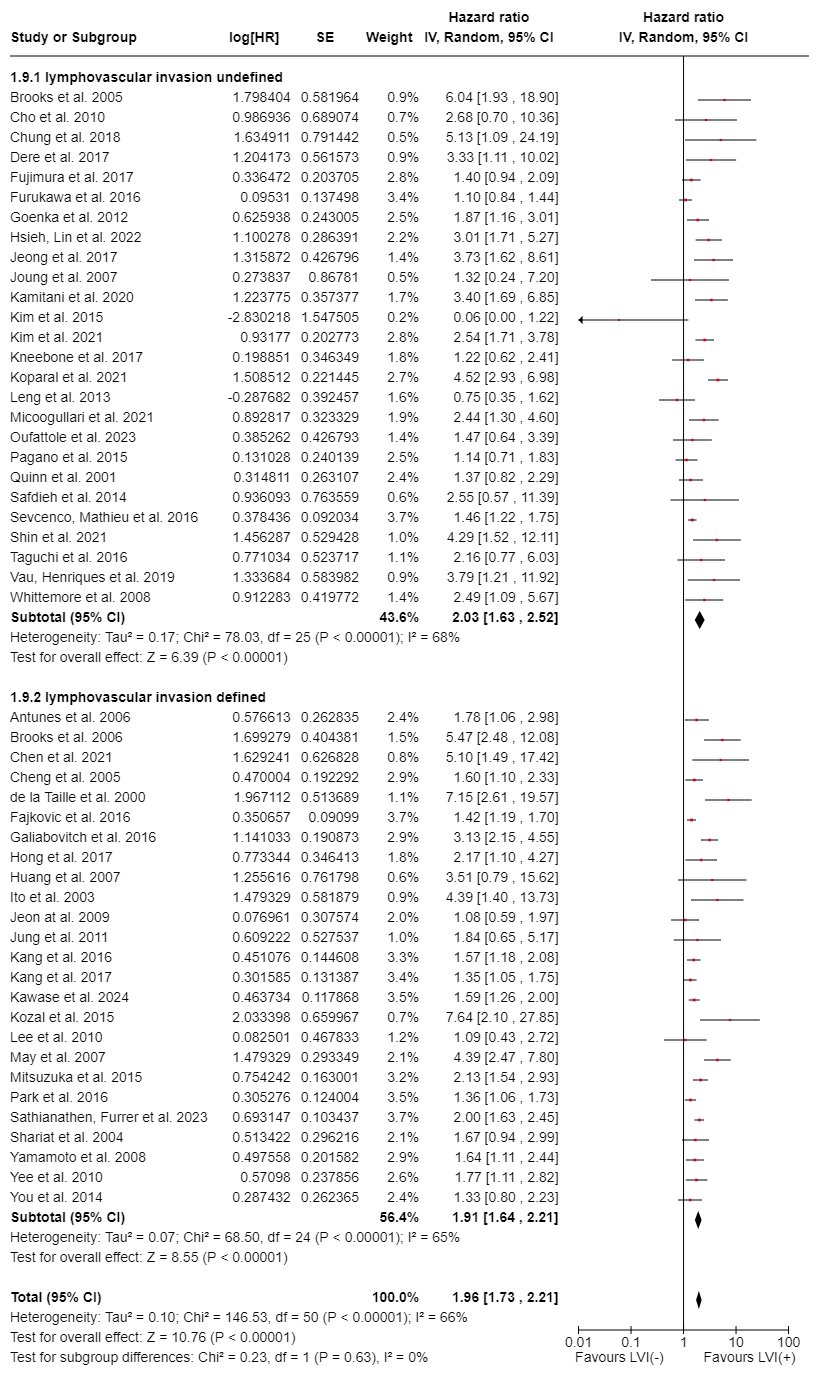


**Supplementary Figure 11.** Forest plot of hazard ratios (HR) in random effects model predicting biochemical recurrence (BCR) according to lymphovascular invasion (LVI) status in subgroups: provided and unprovided definition of LVI. CI = confidence interval; df = degree of freedom; M-H = Mantel–Haenszel; SD = standard deviation.


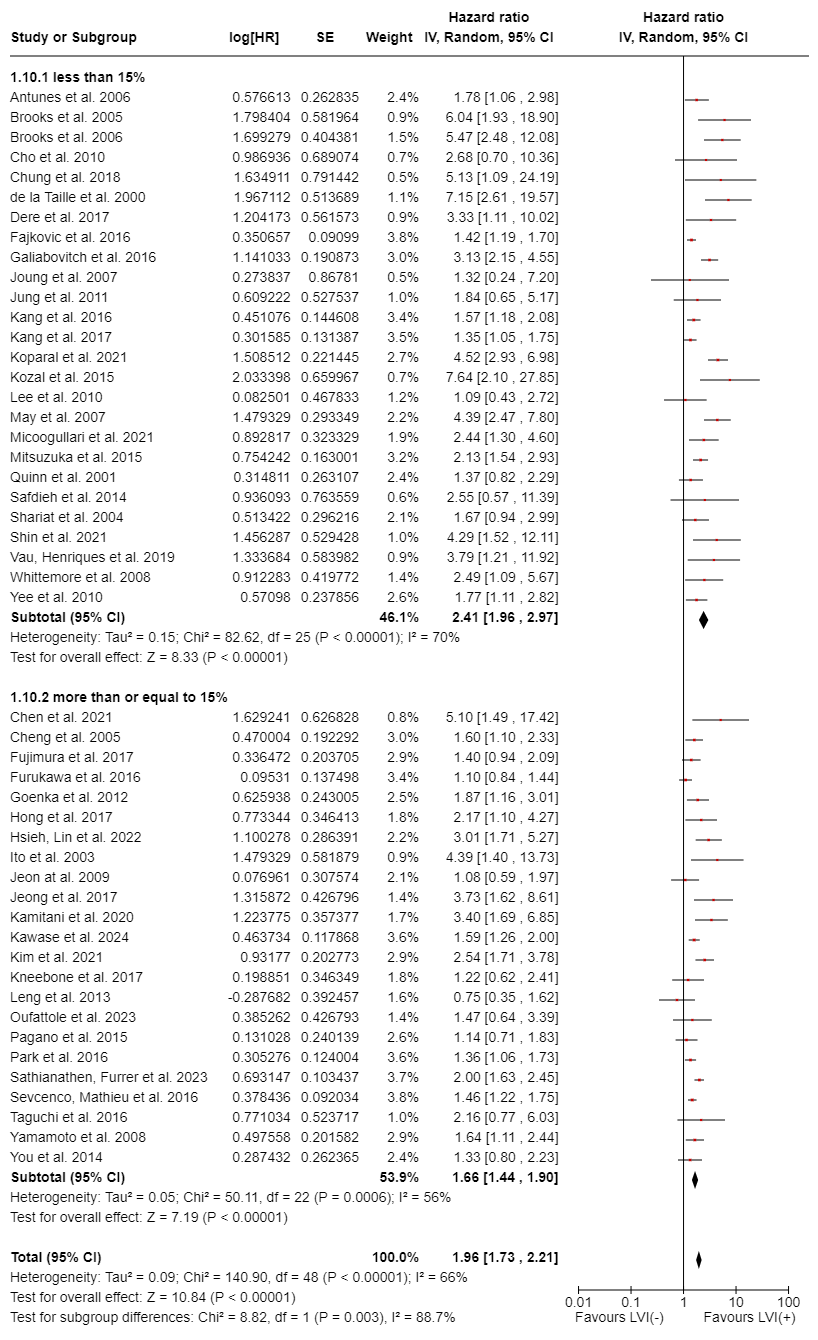


**Supplementary Figure 12.** Forest plot of hazard ratios (HR) in random effects model predicting biochemical recurrence (BCR) according to lymphovascular invasion (LVI) status in subgroups: less than 15% and greater than or equal to 15% LVI-positive men in the patient population. CI = confidence interval; df = degree of freedom; M-H = Mantel–Haenszel; SD = standard deviation.


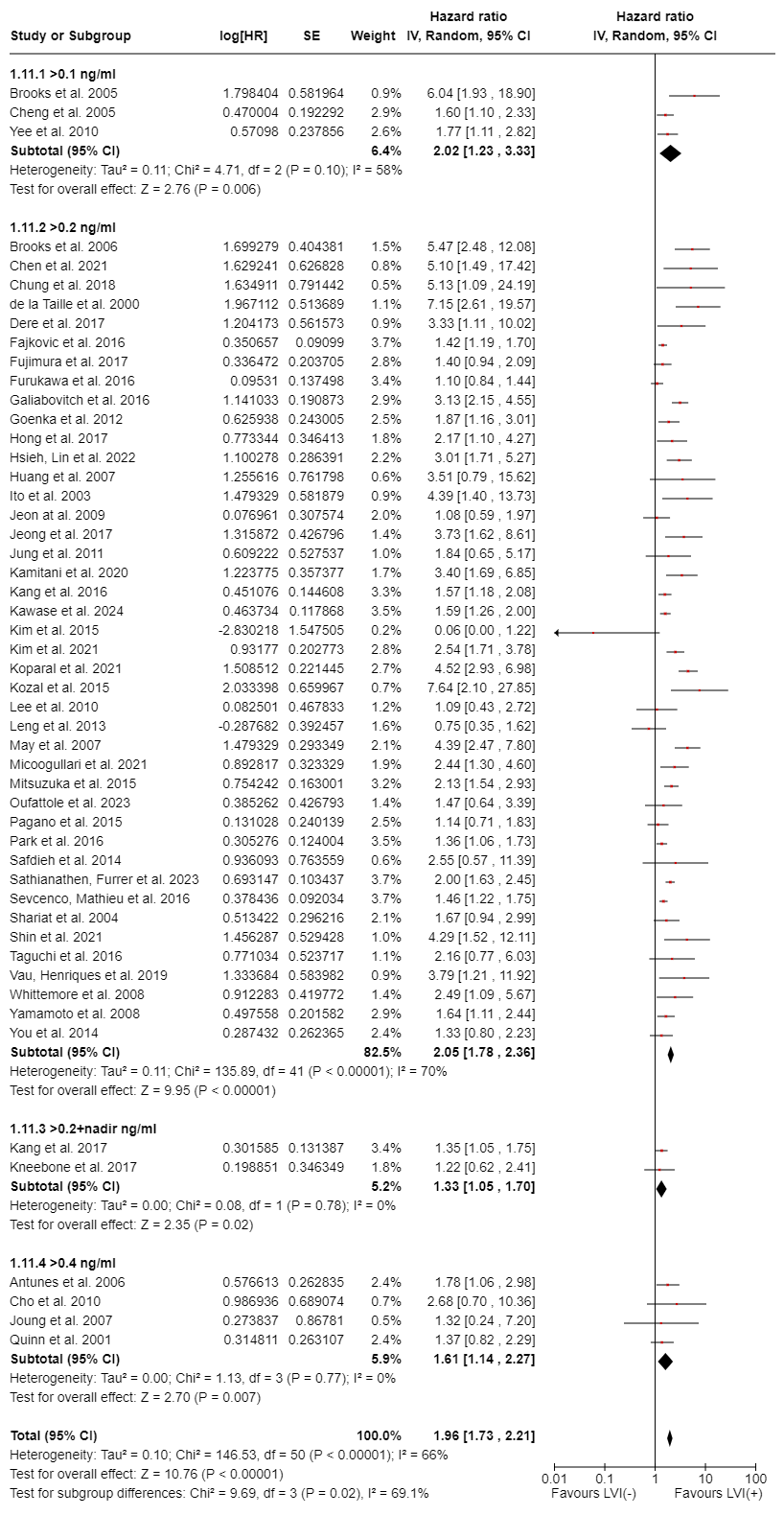


**Supplementary Figure 13.** Forest plot of hazard ratios (HR) in random effects model predicting biochemical recurrence (BCR) according to LVI in subgroups: PSA cutoff value of >0.1 ng/ml, >0.2 ng/ml, >0.2+nadir ng/ml, or >0.4 ng/ml. CI = confidence interval; df = degree of freedom; M-H = Mantel–Haenszel; SD = standard deviation.


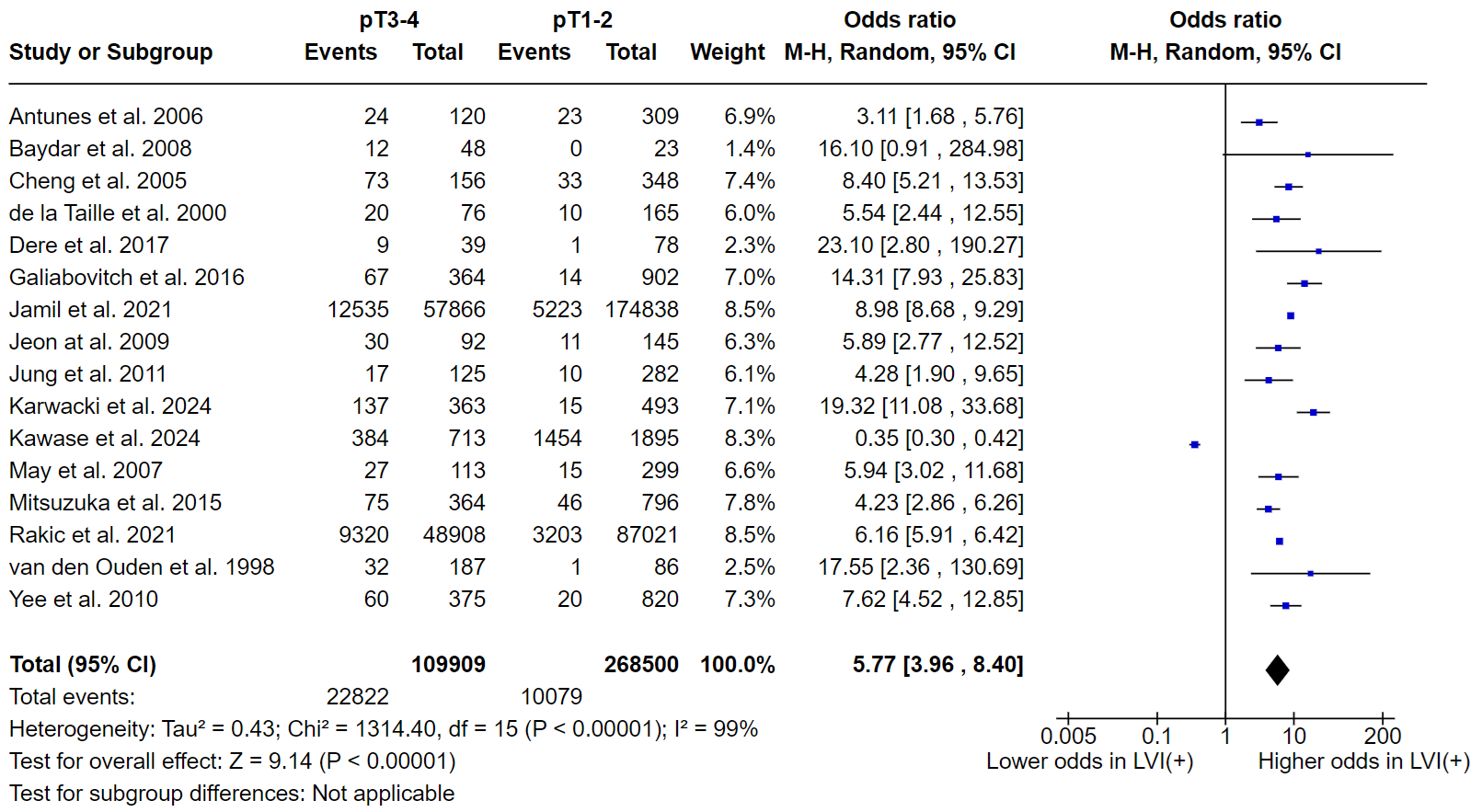


**Supplementary Figure 14.** Forest plot of odds ratios (OR) in random effects model predicting higher pathologic tumor stage (pT) according to lymphovascular invasion status. CI = confidence interval; df = degree of freedom; M-H = Mantel–Haenszel; SD = standard deviation.


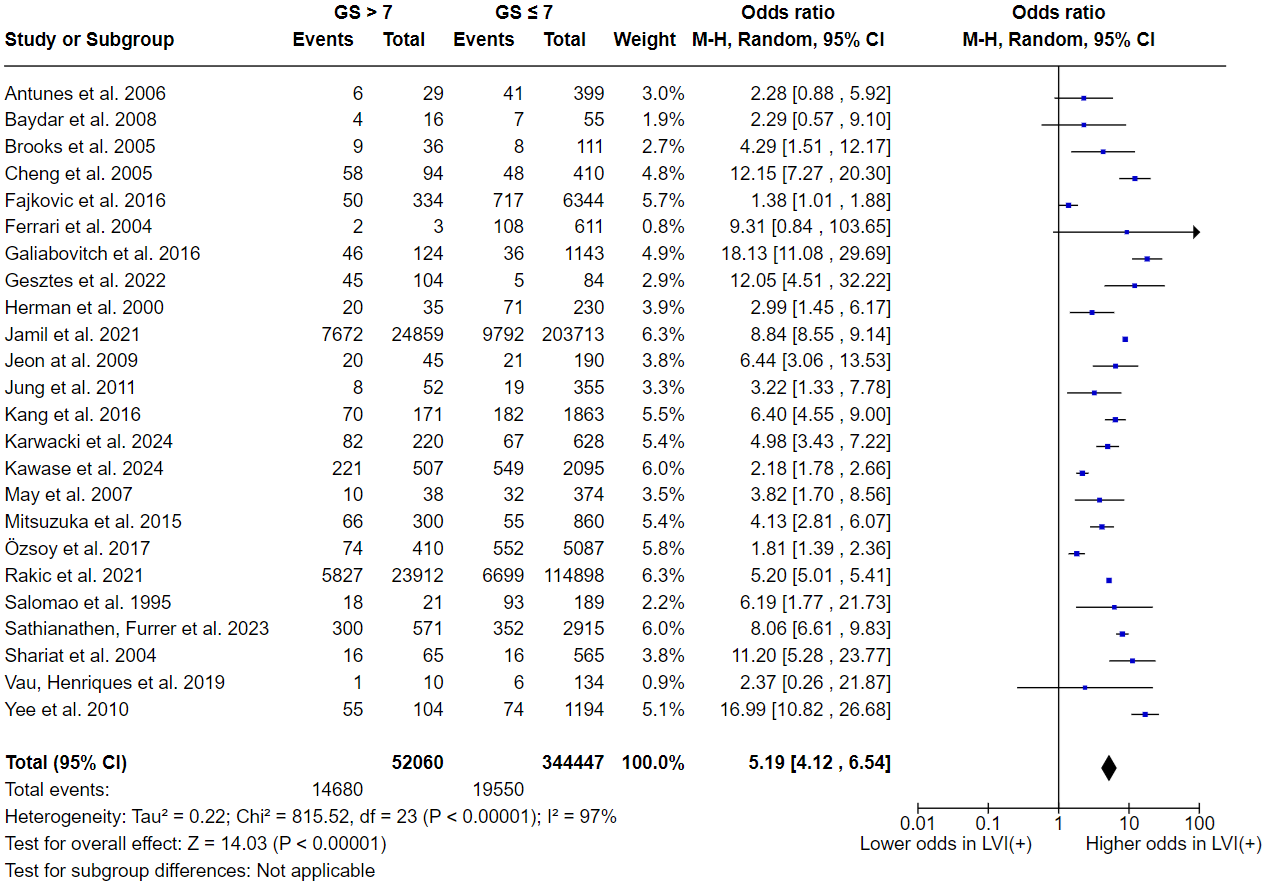


**Supplementary Figure 15.** Forest plot of odds ratios (OR) in random effects model predicting Gleason score according to lymphovascular invasion status. CI = confidence interval; df = degree of freedom; M-H = Mantel–Haenszel; SD = standard deviation.


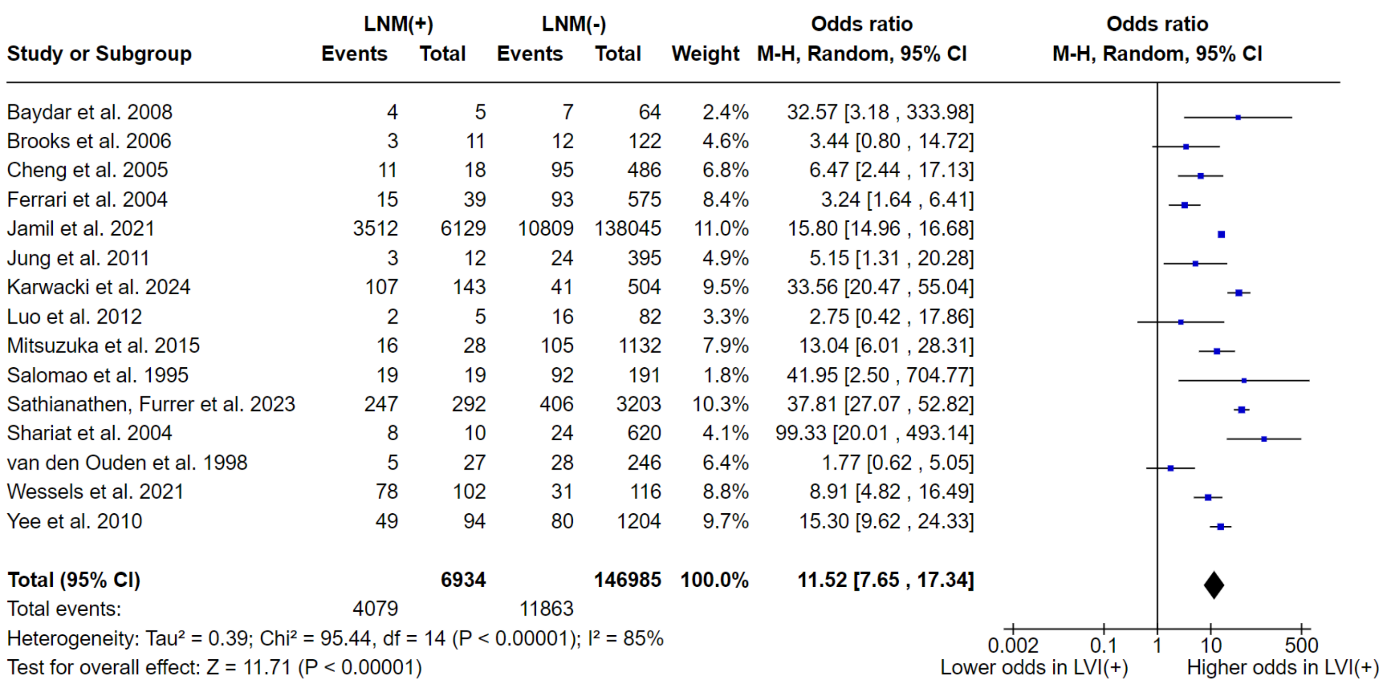


**Supplementary Figure 16.** Forest plot of odds ratios (OR) in random effects model predicting lymph node metastasis according to lymphovascular invasion status. CI = confidence interval; df = degree of freedom; M-H = Mantel–Haenszel; SD = standard deviation.


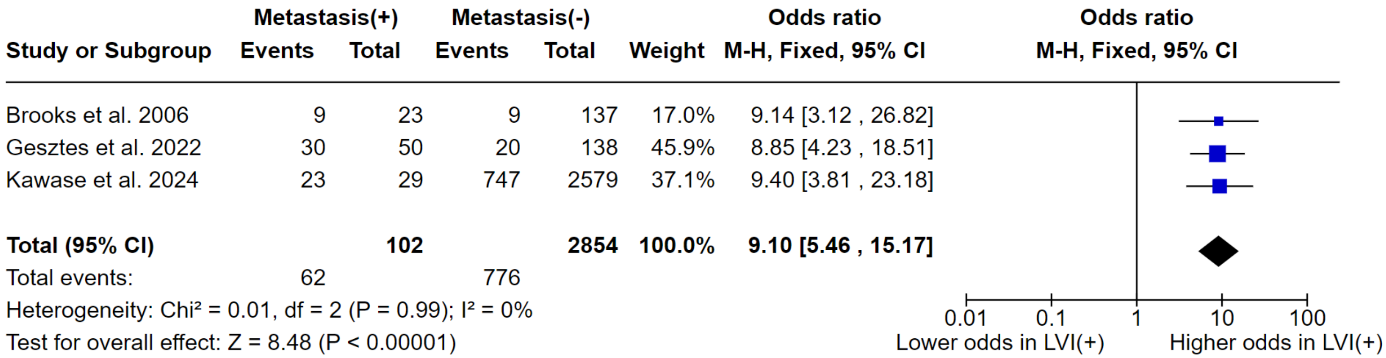


**Supplementary Figure 17.** Forest plot of odds ratios (OR) in fixed effects model predicting distant metastasis according to lymphovascular invasion status. CI = confidence interval; df = degree of freedom; M-H = Mantel–Haenszel; SD = standard deviation.


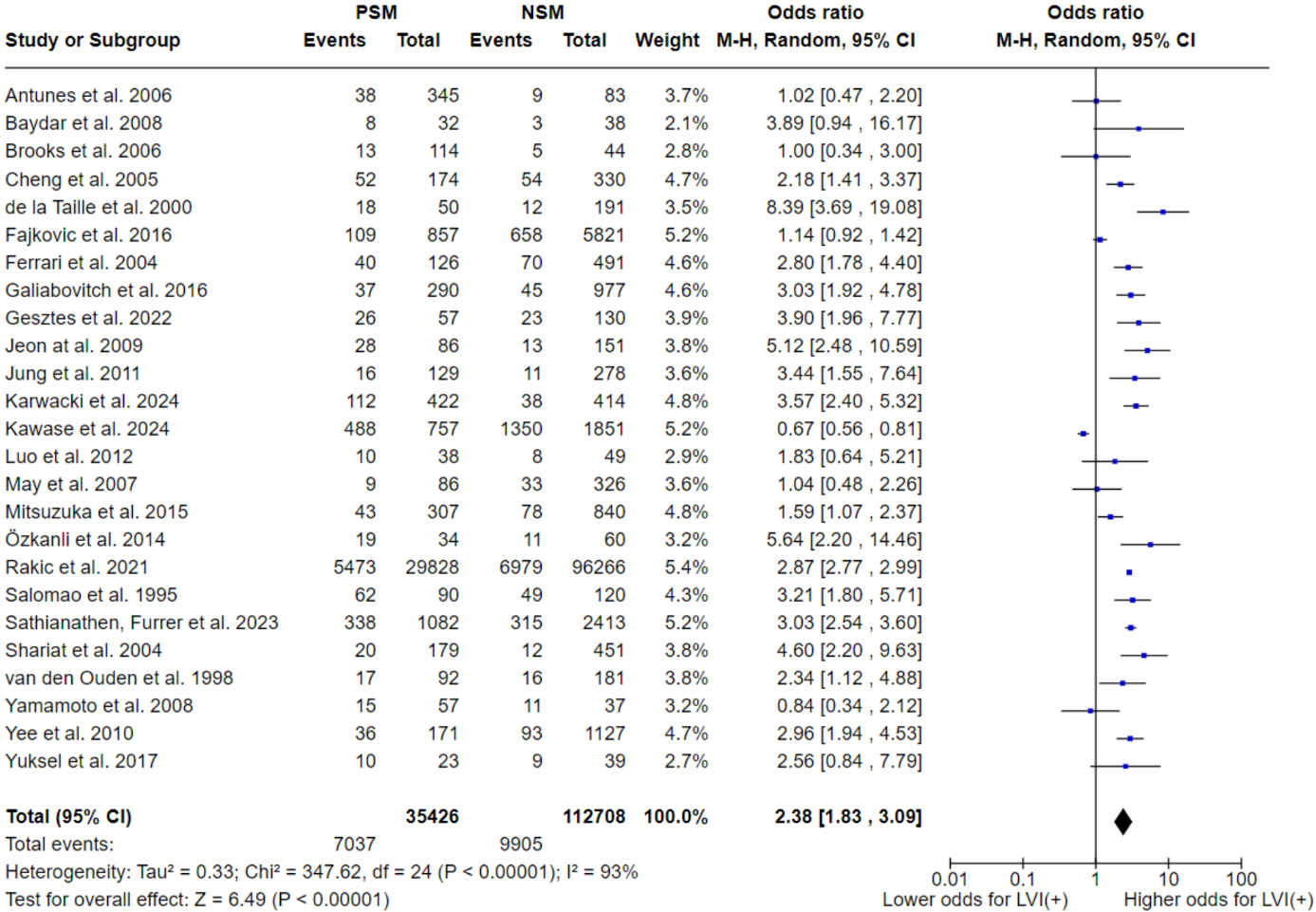


**Supplementary Figure 18.** Forest plot of odds ratios (OR) in random effects model predicting surgical margin status according to lymphovascular invasion status. PSM = positive surgical margin; NSM = negative surgical margin; CI = confidence interval; df = degree of freedom; M-H = Mantel–Haenszel; SD = standard deviation.


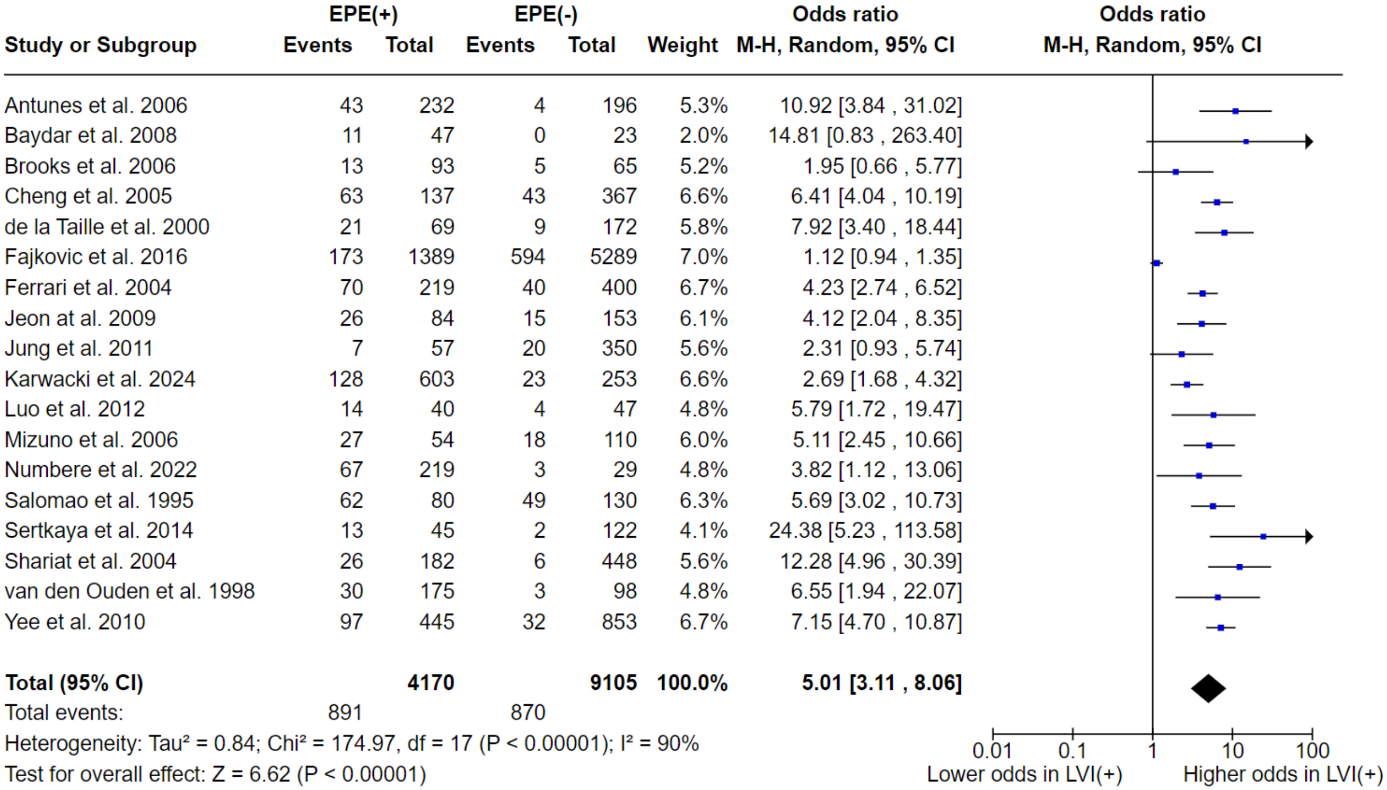


**Supplementary Figure 19.** Forest plot of odds ratios (OR) in random effects model predicting extraprostatic extension (EPE) according to lymphovascular invasion status. CI = confidence interval; df = degree of freedom; M-H = Mantel–Haenszel; SD = standard deviation.


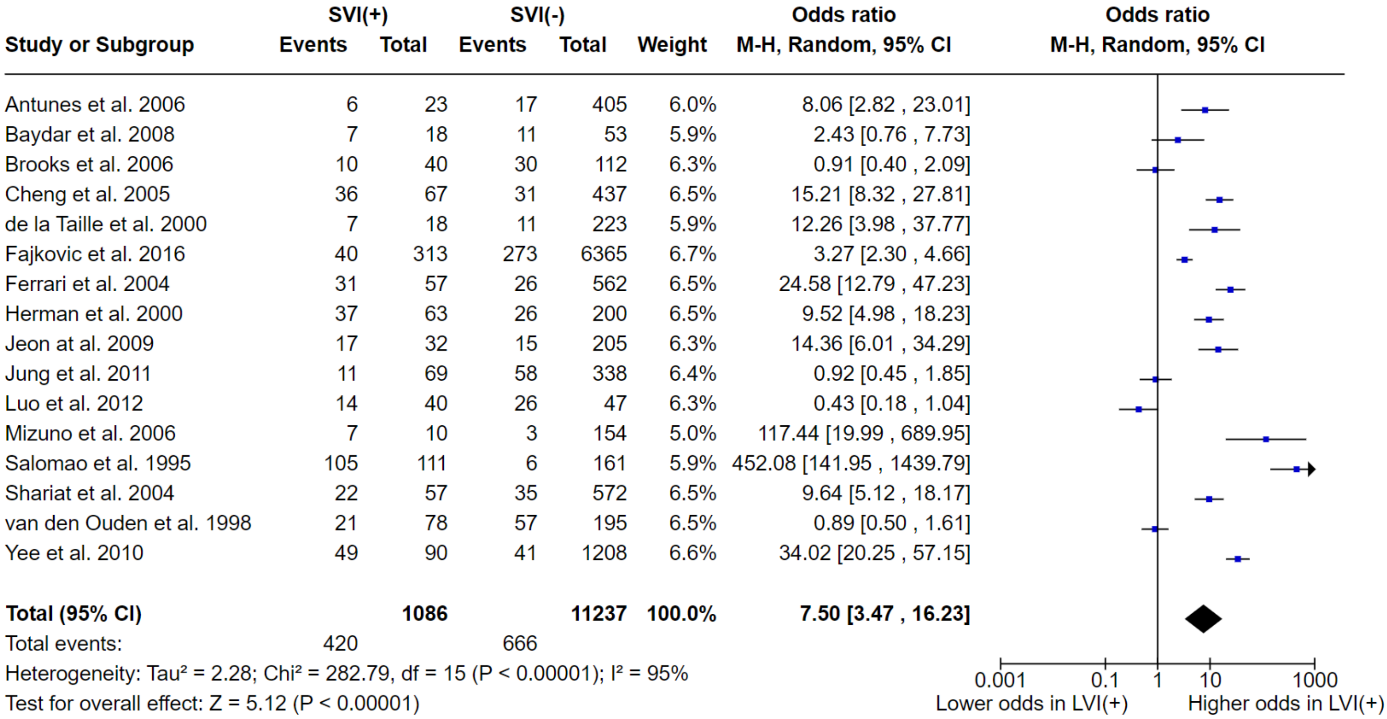


**Supplementary Figure 20.** Forest plot of odds ratios (OR) in random effects model predicting seminal vesicle invasion (SVI) according to lymphovascular invasion status. CI = confidence interval; df = degree of freedom; M-H = Mantel–Haenszel; SD = standard deviation.


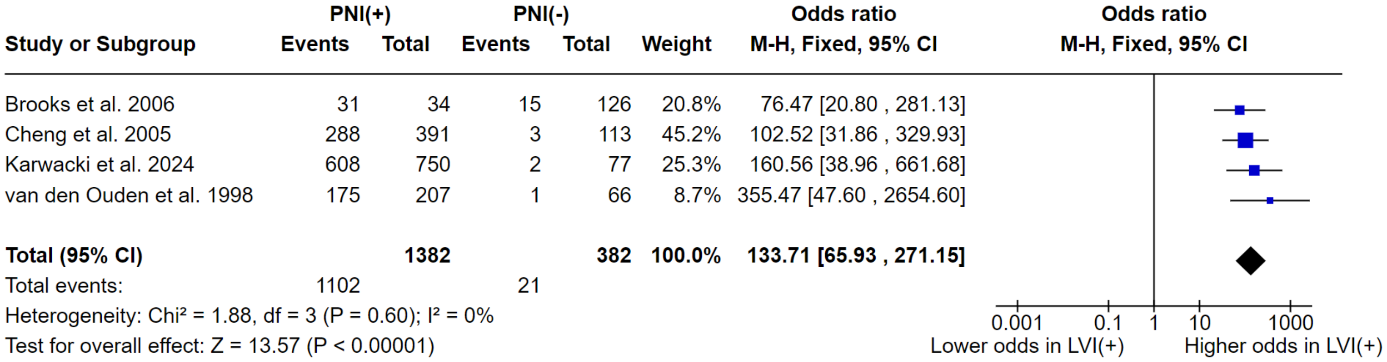


**Supplementary Figure 21.** Forest plot of odds ratios (OR) in fixed effects model predicting perineural invasion (PNI) according to lymphovascular invasion status. CI = confidence interval; df = degree of freedom; M-H = Mantel–Haenszel; SD = standard deviation.


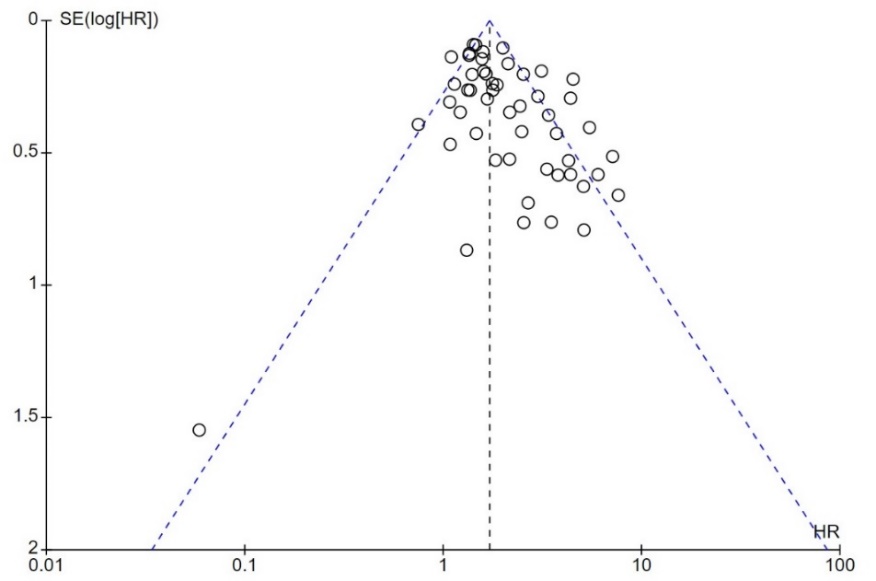


**Supplementary Figure 22.** Funnel plot for the evaluation of potential publication bias comprising 51 studies included in the main meta-analysis on biochemical recurrence with fixed effect model applied. SE = standard error; HR = hazard ratio.
